# miR-455-5p promotes pathological cardiac remodeling via suppression of PRMT1-mediated Notch signaling pathway

**DOI:** 10.1007/s00018-023-04987-2

**Published:** 2023-11-11

**Authors:** Sidong Cai, Junlei Chang, Mengqi Su, Yinxia Wei, Haoran Sun, Cong Chen, Kai-Hang Yiu

**Affiliations:** 1 Division of Cardiology, Department of Medicine, The University of Hong Kong-Shenzhen Hospital, Shenzhen, China; 2 Institute of Biomedicine and Biotechnology, Shenzhen Institute of Advanced Technology, Chinese Academy of Sciences, Shenzhen, China; 3 School of Public Health, Southern Medical University, Guangzhou, China; 4 Department of Clinical Microbiology and Infection Control, The University of Hong Kong-Shenzhen Hospital, Shenzhen, China

**Keywords:** MiR-455-5p, PRMT1, Asymmetric di-methylation, Notch signaling pathway, Pathological cardiac remodeling

## Abstract

Pathological cardiac remodeling plays an essential role in the progression of cardiovascular diseases, and numerous microRNAs have been reported to participate in pathological cardiac remodeling. However, the potential role of microRNA-455-5p (miR-455-5p) in this process remains to be elucidated. In the present study, we focused on clarifying the function and searching the direct target of miR-455-5p, as well as exploring its underlying mechanisms in pathological cardiac remodeling. We found that overexpression of miR-455-5p by transfection of miR-455-5p mimic in vitro or tail vain injection of miR-455-5p agomir in vivo provoked cardiac remodeling, whereas genetic knockdown of miR-455-5p attenuated the isoprenaline-induced cardiac remodeling. Besides, miR-455-5p directly targeted to 3’-untranslated region of protein arginine methyltransferase 1 (PRMT1) and subsequently downregulated PRMT1 level. Furthermore, we found that PRMT1 protected against cardiac hypertrophy and fibrosis in vitro. Mechanistically, miR-455-5p induced cardiac remodeling by downregulating PRMT1-induced asymmetric di-methylation on R1748, R1750, R1751 and R1752 of Notch1, resulting in suppression of recruitment of Presenilin, Notch1 cleavage, NICD releasing and Notch signaling pathway. Finally, circulating miR-455-5p was positively correlated with parameters of left ventricular wall thickening. Taken together, miR-455-5p plays a provocative role in cardiac remodeling via inactivation of the PRMT1-mediated Notch signaling pathway, suggesting miR-455-5p/PRMT1/Notch1 signaling axis as potential therapeutic targets for pathological cardiac remodeling.

**Supplementary Information:**

The online version contains supplementary material available at 10.1007/s00018-023-04987-2.

## Introduction

Pathological cardiac remodeling is a general term which is used to describe a series of abnormal transformations in cardiac structures in response to pathological stimulus (hypertension, myocardial ischemia, valvular stenosis, etc.). Because structural abnormalities gradually emerge in heart during this process, stroke volume and cardiac output is significantly impaired, which leads to cardiac malfunction and loss of ability to meet the demand of the whole body [1]. If treatments fail to stop or delay the development of pathological cardiac remodeling, the heart will continuously deteriorate and eventually progress to heart failure [2–4]. Thus, pathological cardiac remodeling is a crucial process in the evolution of heart failure. Nowadays, in spite of the reduced death rate attribute to the development of medical treatments, survivors still suffer from heart failure due to the complex mechanisms of pathological cardiac remodeling. Therefore, it is of great importance to find new regulatory factors in pathological cardiac remodeling and to elucidate the mechanism therein.

MicroRNAs (miRNAs) were discovered in the early 2000s, consensus indicates that these small noncoding RNAs bind to 3’ untranslated region (3' UTR) of their targeted messenger RNA (mRNA) and suppress expression of targeted mRNA, thus influence many cellular processes [5–7]. In the past two decades, numerous cardiac miRNAs have been implicated in the regulation of cardiovascular diseases [8–10]. Among them, microRNA-455-5p (miR-455-5p) is reported to be involved in cardiovascular diseases such as such as atrial fibrillation, atherosclerosis and hypoxic damage [11–13]. In a prior study, researchers reported that miR-455-5p participated in activation of STAT3, a transcriptional factor which is pivotal in cardiac remodeling [14]. Besides, another research team reported that overexpression of miR-455-5p resulted in hypertrophy of myotubes [15], indicating the role of miR-455-5p in promoting pathological cardiac remodeling is non-negligible. But so far, conclusive evidence for the involvement of miR-455-5p in the progression of pathological cardiac remodeling is lacking.

Protein methylation is the process of transferring one or two methyl groups from S-adenosylmethionine to a nitrogen atom of the guanidine group on an arginine residue. This molecular mechanism was first discovered as a posttranslational modification affecting chromatin remodeling and transcription in histones [16, 17]. Protein methylation is a widespread and evolutionarily conserved posttranslational modification of extra-nuclear proteins [18]. In mammalian cells, protein arginine methyltransferases (PRMTs) regulate protein methylation modification. Of the PRMTs family, protein arginine methyltransferase 1 (PRMT1) is a crucial methyltransferase which is able to mediate asymmetric di-methylation on arginine residues. A recent study indicated that 85% of asymmetric di-methylation was associated with PRMT1 in mammals [19]. Mice of PRMT1-depletion in heart developed severe dilated cardiomyopathy and progressed to heart failure 2 months after birth [20]. Similar result was reported in patient with heart failure, which PRMT1 level was significantly declining in heart tissue. In addition, PRMT1 promoted asymmetric di-methylation of CaMKII at R9 and R275 sites, thus hindered the activation of CaMKII and protected against cardiac hypertrophy and heart failure [21]. Therefore, the roles of PRMT1 and asymmetric di-methylation activity indicate PRMT1 is a potential therapeutic application in treating pathological cardiac remodeling. Nonetheless, it is unclear whether PRMT1 is a target of miR-455-5p to date. In addition, a study reported that myocardial Notch1 guided cardiomyocytes to locate the appropriate spatial position of the ventricular wall. Specific inhibition of cardiac Notch1 activity led to a decrease in ventricular volume and an increase in ventricular wall thickness [22], indicating the important role of Notch1 in pathological cardiac remodeling. However, association between PRMT1 and Notch1 in the heart remains to be revealed.

Therefore, the present study aims to clarify the details of miR-455-5p in regulating PRMT1 expression, determine the function of PRMT1-mediated asymmetric di-methylation of Notch1 in pathological cardiac remodeling, and analyze the underlying mechanisms of interactions in miR-455-5p/PRMT1/Notch1 axis.

## Materials and methods

### Primary cell culture

Neonatal rat cardiomyocytes (NRCMs) and cardiac fibroblasts (NRFBs) were obtained from 1-to 3-day-old Sprague–Dawley (SD) rats according to the previous reported protocol [23]. Briefly, hearts were chopped into small pieces and digested at 37 °C in 0.08% (w/v) trypsin solution for 50–60 min. Cells were collected by centrifugation at 1500*g* for 6 min and re-suspended with Dulbecco's modified Eagle's medium (Gibco, BRL Co, Ltd, USA) containing 10% fetal bovine serum (Cellmax, Beijing, China). To separate NRFBs from NRCMs, cells were seeded in two 25 cm^2^ culture flasks for 1 h at 37 °C incubator containing 5% CO_2_ atmosphere, the supernatant were NRCMs while the adherent cells were NRFBs. Finally, NRCMs and NRFBs were cultured at culture dishes with 60–70% of confluent, respectively. 1% of penicillin–streptomycin solution and 0.1 mM 5-bromodeoxyuridine (5-BrdU) were added into the NRCMs culture medium to prevent contamination and potential fibroblasts proliferation. After 24-hincubation, culture medium was replaced with new medium.

### Animal procedures

#### Ethics and guidelines

All animal studies were conducted in accordance with local institutional guidelines and regulations, including approval for application of animals by Medical Ethics Committee of the University of Hong Kong-Shenzhen Hospital (Serial number: [2022]160). All procedures employed in the experiments were strictly complied with institutional guidelines for the Guide for the Care and Use of Laboratory Animals [24, 25].

#### Allocations

Briefly, 18–22 g C57BL/6 male mice (No. 44822700005168) in SPF grade were purchased from Zhuhai BesTest Bio-Tech Co., Ltd (Zhuhai, China). Mice were housed in specific pathogen free (SPF) environment with 12:12 h light/dark cycle, room temperature was between 21 and 23 °C. All mice had free access to standard laboratory food and water and were given 1 week to acclimatize new environment before the experiments. For allocations, all of the 36 mice were randomly and equally divided into 6 groups, namely NC agomir group, miR-455-5p agomir group, NC antagomir group, NC antagomir + isoprenaline (ISO) group, miR-455-5p antagomir group and miR-455-5p antagomir + ISO group, respectively. The schemas illustrating the timeline of the principal experimental procedures in mice are presented in Supplementary Figure (Fig. S1).

#### ISO administration

Isoproterenol hydrochloride (T1056, TargetMol) as a crystalline powder and was solubilized in NaCl 0.9% according to the manufacturer’s guidelines. To induce pathological cardiac remodeling model, mice in NC antagomir + ISO group and NC antagomir + ISO group were subcutaneously administrated ISO (1.5 mg/kg/d) between day 19 and day 28 of the experiment, while the mice in the rest 4 groups were administrated the same volume of saline.

#### miR-agomir and miR-antagomir administration

Generally, miR-455-5p agomir was synthetized and purchased from RibBio Ltd. (Guangzhou, Guangdong, China). The miR-455-5p agomir was an oligonucleotide to mimic the mature sequence of miR-455-5p. The scrambled control sequence (NC agomir) that contained the same amount of bases was regarded as negative control. The miR-455-5p agomir or an equal dose of NC agomir was injected via tail vain at day 1, 4, 8, 11, 15, 18, 22 and 25. The dose of each injection was 10 nmol, miR-455-5p agomir and NC agomir were solubilized in 0.9% saline.

miR-455-5p antagomir for in vivo applications was synthetized and purchased from Guangzhou RiboBio Co., Ltd. The miR-455-5p antagomir was an oligonucleotide directed against the mature sequence of miR-455-5p and was modified with locked nucleic acid technology to ensure stability. The scrambled control sequence (NC antagomir) contained the same amount of building blocks (ACGT) but in a random order. MiR-455-5p antagomir or NC antagomir was injected via tail vain at day 1, 4, 8, 11, 15, 18, 22 and 25. The dose of each injection was 20 nmol, both miR-455-5p antagomir and NC antagomir were solubilized in 0.9% saline.

### Echocardiography

Mice were anesthetized by 0.5–1.0% isoflurane in 100% oxygen and placed on a heated platform for echocardiography. Echocardiography in two-dimensionally guided M-mode was performed to evaluate the left ventricular function via a Vevo 2100 echocardiography system (VisualSonics, Toronto, Canada). Basic cardiac function parameters, such as EF (ejection fraction), IVST (interventricular septum), LVPW (left ventricular posterior wall) and LVID (left ventricular internal dimension) were recorded for following statistical analysis.

### Histology

#### Sample preparation

Mice were anesthetized and sacrificed by intraperitoneal injection of overdosed sodium pentobarbital. Hearts were arrested in diastole state by means of potassium chloride buffer and were rapidly transferred to ice-cold phosphate buffer (PBS). Then, each heart was trimmed in transverse plane and was separated into two parts. One part was fixed with 4% paraformaldehyde and embedded in paraffin for hematoxylin–eosin (H&E) staining, Masson staining and wheat germ agglutinin (WGA) staining, the other part was kept in liquid nitrogen and then preserved in − 80 °C environment for further mRNA and protein analysis.

#### Histological staining

Heart tissue embedded in paraffin was cut into 4- to 5-μm sections. Paraffin sections were stained with H&E for routine histological analysis, Masson for detection of collagen synthesis, WGA staining for detection of cell surface area of NRCMs in heart. Slides were visualized using a Zeiss Axio Vert.A1 Imager microscope (CarlZeiss Inc.).

### Western blotting

Total protein was extracted by using RIPA lysis buffer (Beyotime, Nantong, Jiangsu, China). Nuclear protein was acquired according to Nuclear Extract Kit (Motif Active, Carlsbad, CA, USA). Subsequently, equal amount of protein samples, together with 5 × SDS loading buffer (Beyotime, Nantong, Jiangsu, China), were mixed and loaded to 8–15% sodium dodecyl sulfate–polyacrylamide gels for electrophoresis. Then, protein on gels were transferred to methanol-presoaked PVDF (polyvinylidene fluoride) membranes (EMD Millipore Corporation, Billerica, MA, USA) under the constant current of 230 mA for 110 min. Membranes were blocked with 5% non-fat milk for 1 h, incubated with primary antibodies (4 °C overnight) and horseradish peroxidase (HRP)-conjugated secondary antibodies (room temperature, 1 h) before detection of blots. By incubation with Tanon™ High-sig ECL Western Blotting Substrate (Tanon, Shanghai, China), blots could be detected by ChemiDoc MP system (BIO-RAD, California, United States). Image J software was utilized to calculated the intensity of blots in different lanes. GAPDH was used as internal control of total protein; Lamin B was used as internal control of nuclear protein. Antibodies were shown in Supplementary Table S1.

### Real-time quantitative polymerase chain reaction (Q-PCR)

RNA from NRCMs, NRFBs and heart tissue was extracted with the Trizol Reagent (Invitrogen, Carlsbad, CA, USA) according to the manufacturer’s instructions. In order to ensure the feasibility of the protocol of RNA extraction, the purity, concentrations, integrity and contamination of the extracted RNA samples is necessary to be examined. For analyzing the purity and concentrations of RNA samples, only when OD260/OD280 is between 1.8 and 2.0, OD260/230 is over 2.0 and the concentration is more than 700 ng/μl, the sample is acceptable for pure RNA. For analyzing the integrity and contamination of RNA samples, RNA samples whose 28S and 18S bands are clear and sharp by denaturing agarose gel electrophoresis are considered as intact and uncontaminated. The RNA samples in this study met the criteria mentioned above, the results are demonstrated in Fig. S2. Then, for mRNA, approximately 2 μg total RNA was reverse-transcribed into first strand cDNA using the RevertAid First Strand cDNA Synthesis Kit (Thermo Fisher Scientific, MA, USA). For miRNA, 2 μg total miRNA was reverse-transcribed to first strand cDNA according to the instructions of miRNA 1st strand cDNA synthesis kit (Accurate Biotechnology, Shenzhen, China). Q-PCR was performed on Roche LightCycler® 480 Real-Time PCR System (Roche), THUNDERBIRD™ SYBR qPCR Mix (Toyobo, Osaka, Japan) was utilized as fluorescent dye. Transcript quantities were compared using the relative Ct method, where the amount of target normalized to the amount of endogenous control (β-actin for mRNA and U6 for miRNA) and relative to the control sample is given by 2^−∆∆Ct^. Primers of mRNA are shown in Supplementary Table S2.

### Measurement of cell surface area

NRCMs cultured in 24-well plates were fixed with paraformaldehyde diluted in PBS (4%, w/v) for 15 min at ambient temperature, followed by 0.3% Triton-100 treatment for 10 min. Then, NRCMs were incubated with 0.1% (v/v) rhodamine-phalloidin in dark for 1 h, and were further stained with DAPI before rinsing NRCMs by PBS. Images of the NRCMs were detected via High Content Screening system (ArrayScanVTI, Thermo Fisher Scientific, Rockford, IL, United States). The cell surface area from randomly selected fields (3 for each group) was determined using the Image J analysis software. Data were presented as fold change to control group.

### Small interference RNA and plasmid transfection

Small interference RNAs (si-RNA) target to PRMT1, together with negative control si-RNA (si-NC), miR-455-5p mimic and inhibitor, were purchased from RibBio, Ltd (Guangzhou, Guangdong, China). Briefly, cells at 60% confluent (6-well) were cultured in Opti-MEM® I Reduced-Serum Medium (Gibco, Grand Island, NY, USA) with mixture containing 5 μl lipofectamine 2000 (Invitrogen, Carlsbad, CA, USA) and 40 nmol siRNA/40 nmol miR-455-5p mimic/80 nmol miR-455-5p inhibitor.

PRMT1 plasmid was cloned into pcDNA3.1( +) vector by VIC GENE (Guangzhou, China). Both wild-type and mutant-type Notch1 plasmid was cloned into pCMV vector by Shhebio (Shanghai, China). In general, 2 μg of plasmid was transfected into NRCMs in 6-well plate via the same way as si-RNA transfection. 4 to 6 h after transfection, culture medium was replaced with fresh DMEM with 10% of fetal bovine serum. Cells were ready for subsequent procedure after 48 to 96 h.

### Co-immunoprecipitation (Co-IP)

Cells or tissue were lysed by IP lysis buffer (Nantong, Jiangsu, China). Approximately 500 μg protein lysate was used for immunoprecipitation, and 10 μg of the lysate was utilized as input. Protein lysate was separately incubated with Notch1 primary antibody (GeneTex, USA) or anti-mouse IgG antibody (Beyotime, Nantong, Jiangsu, China) overnight at 4 °C. Then, 30 μl protein A/G beads (MedChemExpress, USA) was added to protein lysate and rotated at 4 °C for 2 h. After washed by washing buffer, beads were collected and boiled with 30 μl of 2 × loading buffer before western blotting assay.

### Target prediction of miR-455-5p

Putative miR-455-5p target genes were identified using target prediction tools TargetScan (http://targetscan.org/index.html).

### Dual-luciferase reporter assay

Dual luciferase reporter vectors containing wild-type (WT) or mutant (Mut) PRMT1 3’UTR were constructed and were transfected into NRCMs. At the same time, NRCMs were further transfected with miR-455-5p mimic/NC mimic or miR-455-5p inhibitor/NC inhibitor, respectively. 48 h later, renilla luciferase activity and luciferase activity was detected by Dual-Luciferase Reporter Assay System (Promega) according manufacturer’s protocols. The results were presented as relative luciferase activity.

### RNA-seq

48 h after transfection with si-NC or si-PRMT1, the NRCMs were collected under RNase-free conditions. Total RNA was isolated from the NRCMs by using TRIzol Reagent (Thermo Fisher Scientific) in accordance with the manufacturer’s protocol. Total RNA (1 μg per sample) was used to construct sequencing libraries. Briefly, mRNA was enriched and cleaved into short fragments by using fragmentation buffer, followed by reverse transcription into cDNA using random primers. The cDNA fragments were purified and end repaired, and a poly(A) tail was added, followed by ligation with Illumina sequencing adapters. The ligation products were then size-selected by agarose gel electrophoresis, amplified by PCR, and sequenced using the Illumina Novaseq 6000 (Gene Denovo Biotechnology). Analysis of differential RNA expression between si-PRMT1 group and si-NC group was conducted by differential expression genes analysis (DEG analysis). Transcripts with the parameter of an adjusted *P* value < 0.05 and an absolute fold change ≥ 1.5 were considered differentially expressed. Pathway enrichment analysis was performed using the KEGG. Adjusted p value of equal or less than 0.05 was defined as significantly enriched pathways.

### Research on circulating miR-455-5p

#### Criteria of study subjects and data collection

The retrospective study was conducted based on hypertensive patients and their available medical data and serum samples. Besides, the study was approved by the Ethics Committee of the University of Hong Kong-Shenzhen Hospital (No: [2022]134) and was performed in accordance with the ethical standards as laid down in the 1964 Declaration of Helsinki. Basically, 46 patients met the diagnostic criteria of hypertensive heart disease and recorded in medical record database of the Department of Cardiology, the University of Hong Kong-Shenzhen Hospital were selected as study subjects.

Hypertensive heart disease was defined using the International Classification of Diseases, Ninth and Tenth Revision (ICD-10) codes. Disease coded as I11.0 and I11.9 in ICD-10 was identified as hypertensive heart disease [26, 27]. Briefly, the hypertensive heart disease cases were selected as long as one of the following criteria is met: 1. Longstanding systolic blood pressure ≥ 140 mmHg and (or) diastolic blood pressure ≥ 90 mmHg (over 1 year); 2. Elevated T segment; 3. Left ventricular wall and interventricular septum thickening presented in echocardiography.

Clinical characteristics were extracted from medical records, including age, sex, body mass index (BMI), medical history (hypertension, diabetes, coronary artery diseases, valvular heart disease), and echocardiographic data (LVEF, LVIDd, IVSTd, LVPWd and E/A).

#### Serum sample preparation and analysis

Serum samples were extracted from blood sample reserved in the Blood Sample Biorepository of the Department of Cardiology, the University of Hong Kong-Shenzhen Hospital. To obtain plasma samples, the blood was centrifuged at 1500*g* for 15 min, followed by centrifugation at 13,000*g* for 2 min. miRNA were extracted and analyzed according to manufacturer’s instructions (Changzhou Bio-generating Biotechnology Corp, China). Repeated freeze-and-thaw cycles were avoided.

### Statistical analysis

For results in animals and cells, data were shown as means ± SEM, and was analyzed by two-tailed unpaired Student’s *t* test between two groups. For comparisons among multiple groups, one-way ANOVA analysis and the Bonferroni post hoc test method were employed. Statistical analysis was accomplished by GraphPad Prism Software Version 5.01(La Jolla, CA). *P* < 0.05 was regarded to be statistically significant.

For results on clinical research, Spearman’s correlation analysis was employed to analyze the correlation between miR-455-5p level and echocardiographic parameters, including LVPWd, IVSTd, relative wall thickness (RWT) and left ventricular mass index (LVMi). *P* < 0.05 was regarded to be statistically significant; *R* ≥ 0.6 was regarded as strong positive correlation. Receiver-operating characteristic curve (ROC) analysis was performed to assess the diagnostic and prognostic value of miR-455-5p in patients with three different cardiac geometry patterns during cardiac remodeling. *P* < 0.05 was regarded as statistically significant; area under curve (AUC) indicates the predictive effect of circulating miR-455-5p. Univariable logistic regression analysis and multivariable logistic regression analysis were applied to study identify factors that were independently associated with diagnosis of concentric left ventricular hypertrophy. The *χ*^2^ test was used for analyzing categorical data. Statistical analysis was performed with IBM SPSS Statistics version 20 (IBM Inc).

### Reagents and drugs

Reagents, drugs, si-RNAs, plasmids and other materials utilized in this study are listed in supplementary document Supplementary Table S3-S5.

## Results

### MiR-455-5p is upregulated in cardiomyocytes and promotes cardiac hypertrophy and cardiac fibrosis in vitro

**Fig. 1:**
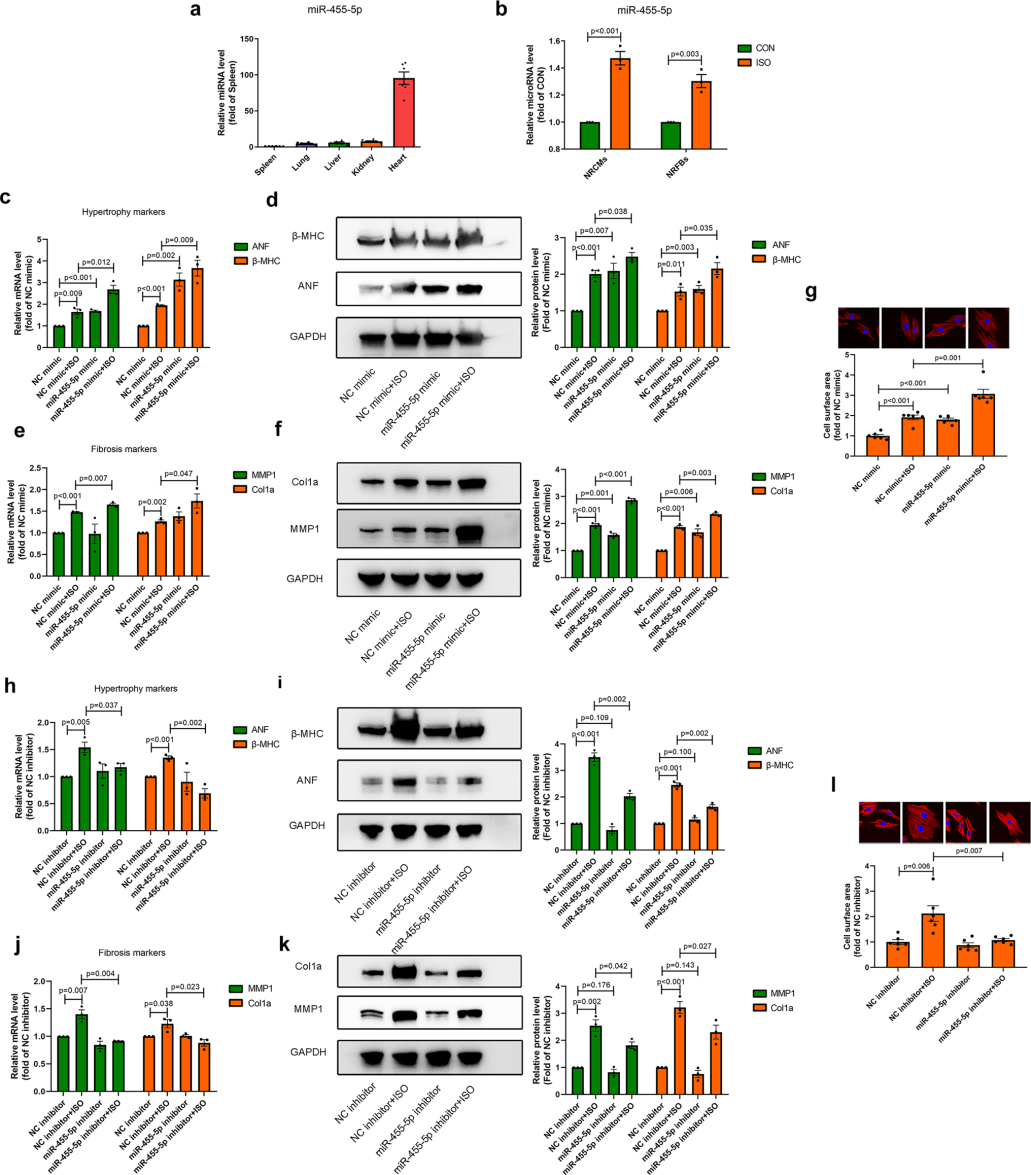
miR-455-5p promotes cardiac hypertrophy and cardiac fibrosis in vitro. **a** mRNA level of miR-455-5p in spleen, lung, liver, kidney and heart are shown (*n* = 6). **b** q-PCR showing the levels of miR-455-5p in NRCMs and NRFBs treated with or without 10 μM ISO for 12 h (*n* = 3). **c–g** q-PCR and western blotting showing the mRNA levels and protein levels of ANF, β-MHC (**c**, **d**), MMP1, Col1a (**e**, **f**), and cell surface area (**g**) measured by rhodamine-phalloidin staining in NRCMs transfected with negative control mimic (NC mimic) or miR-455-5p mimic in the presence or absence of ISO (*n* = 3). **h**–**l** q-PCR and western blotting showing the mRNA levels and protein level of ANF, β-MHC (**h**, **i**), MMP1, Col1a (**j**, **k**), and cell surface area (**l**) measured by rhodamine-phalloidin staining in NRCMs transfected with negative control inhibitor (NC inhibitor) or miR-455-5p inhibitor in the presence or absence of ISO (*n* = 3)

To investigate the baseline abundance of miR-455-5p in different organs, C57BL/6 mice without receiving any pharmacological or surgical treatments were sacrificed. Spleens, lungs, livers, kidneys and hearts were harvested to determine the level of miR-455-5p by q-PCR assay. Compared to the rest four organs, miR-455-5p level in heart was much higher, indicating that miR-455-5p may play an important role in heart (Fig. 1a). It is acknowledged that cardiac hypertrophy and cardiac fibrosis are two key phenotypes of pathological cardiac remodeling, and isoprenaline (ISO) can be employed to induce pathological cardiac remodeling in vitro [28–30]. Thus, we utilized ISO to construct cardiac hypertrophy model in NRCMs and cardiac fibrosis model in NRFBs. As a result, a significant increase in miR-455-5p level was observed both in NRCMs and NRFBs treated with ISO (Fig. 1b). Thus, miR-455-5p was upregulated in the development of pathological cardiac remodeling.

Next, to explore the function of miR-455-5p in cardiac hypertrophy and cardiac fibrosis in vitro, miR-455-5p was knocked-down and over-expressed by validated miR-455-5p inhibitor and miR-455-5p mimic (Fig. S3a, b). At baseline (without ISO stimulus), miR-455-5p mimic not only increased the levels of hypertrophic markers and cell surface area in NRCMs, but also elevated the levels of fibrosis markers in NRFBs. Furthermore, miR-455-5p further aggravated these phenotypes in the presence of ISO (Fig. 1c–g). Meanwhile, treatment of miR-455-5p inhibitor blunted ISO-mediated hypertrophic and fibrosis responses (Fig. 1h–l).

Taken together, these results suggest that miR-455-5p promotes cardiac hypertrophy and cardiac fibrosis in vitro.

### Manipulation of miR-455-5p level in mice affects the pathological cardiac remodeling in vivo

**Fig. 2:**
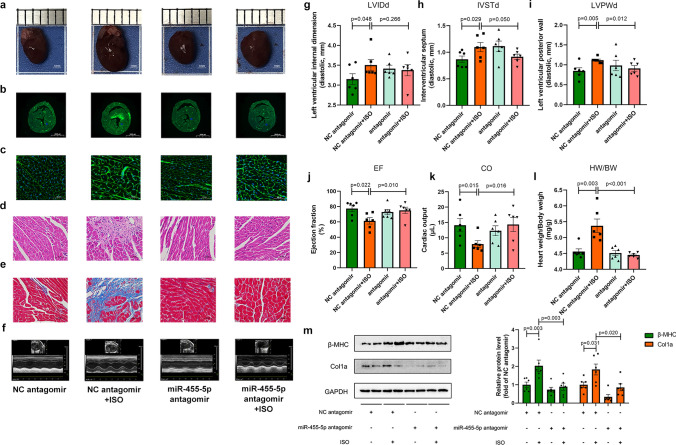
miR-455-5p antagomir protects heart from cardiac remodeling in vivo. **a** Representative gross morphology are shown, scale bar is 1 mm. **b**, **c** WGA staining of cross-section and their 40 × amplification are shown, scale bar is 2000 μm in **b** and is 50 μm in **c**. **d** H&E staining of the hearts are shown, scale bar is 50 μm. **e** Masson’s staining of the hearts are shown, scale bar is 50 μm. **f** Echocardiographic analysis are shown. **g** The left ventricular internal diameter, **h** interventricular septum, **i** left ventricular posterior wall, **j** ejection fraction, **k** cardiac output and **l** heart weight versus body weight are measured (*n* = 6). **M** Hypertrophy markers β-MHC and fibrosis marker Col1a, are measured by western blotting analysis (*n* = 6)

ISO is an effective reagent to induce pressure overload, and cardiac pressure overload is a common factor to provoke pathological cardiac remodeling [28], therefore, we chose ISO to construct cardiac remodeling model in vivo. Then, to figure out the level of miR-455-5p in vivo, mice were injected with specific oligonucleotides against miR-455-5p (miR-455-5p antagomir) or negative control oligonucleotides (NC antagomir) via tail vein. The workflows of the procedures about animal experiments are presented in Fig. S1a. The efficiency of miR-455-5p antagomir in mice heart tissue were validated (Fig. S3c). As a result, cardiac morphology, the size of cardiomyocytes in heart, hypertrophic and fibrotic responses significantly increased after 10 days of ISO in NC antagomir mice, whereas mice injected with miR-455-5p antagomir blunted these responses (Fig. 2a–e). Furthermore, echocardiography demonstrated that abnormal cardiac structure (increasing IVSTd and LVPWd), impaired cardiac function (EF and CO) and elevated heart weight/body weight ratio (HW/BW) emerged in mice administrated with ISO, but these phenotypes were alleviated by injection with miR-455-5p antagomir (Fig. 2f–l). Moreover, β-myosin heavy chain (β-MHC) and collagen 1a (Col1a)—the markers indicate cardiac hypertrophy and cardiac fibrosis—were upregulated in mice administrated with NC antagomir and ISO, whereas they were restored in mice treated with miR-455-5p antagomir and ISO (Fig. 2m).

**Fig. 3:**
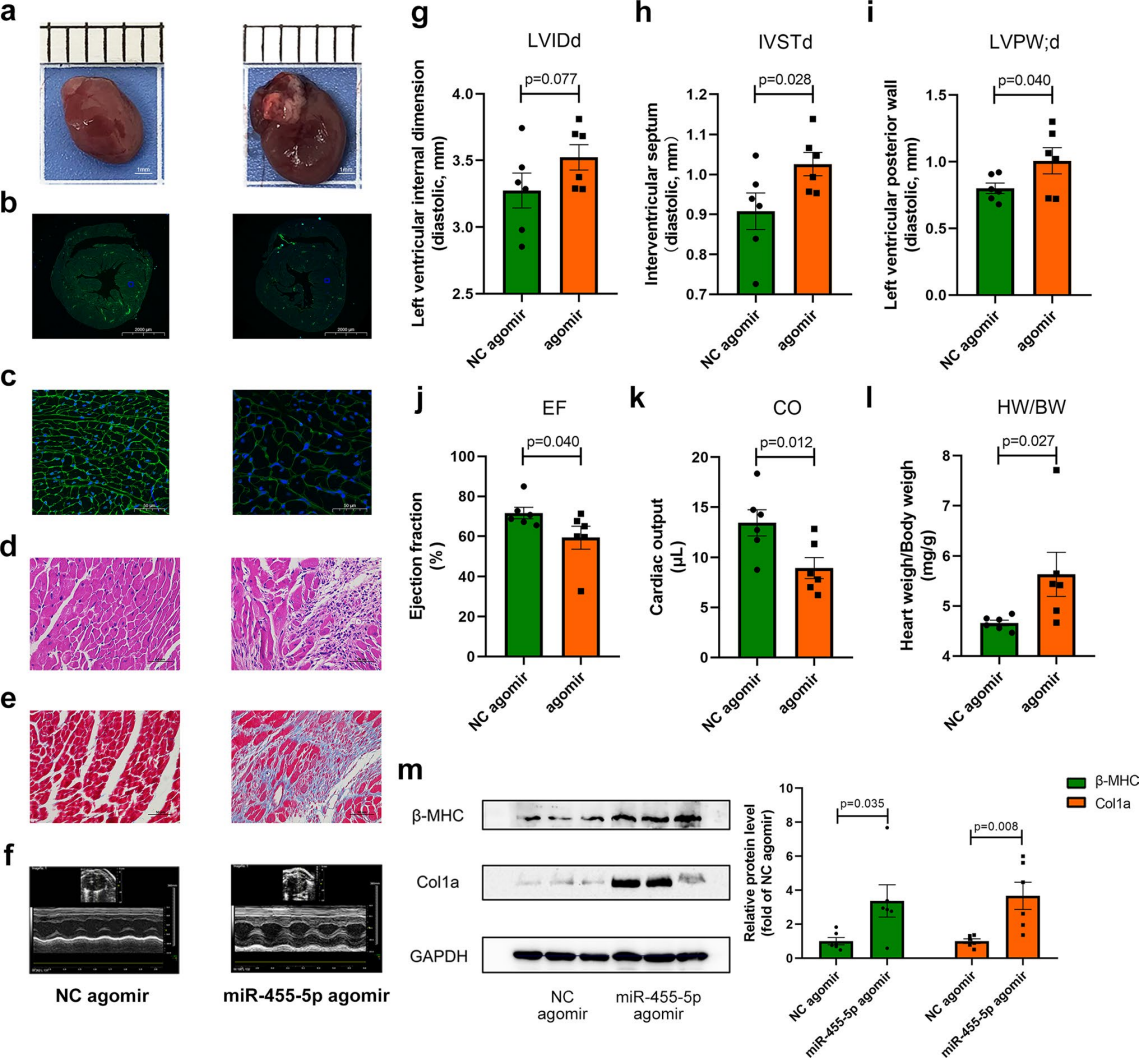
miR-455-5p agomir induces cardiac remodeling in vivo. **a** Representative gross morphology are shown, scale bar is 1 mm. **b, c** WGA staining of cross-section (**b**) and their 40 × amplification (**c**) are shown, scale bar is 2000 μm in **b** and is 50 μm in **c**. **d** H&E staining of the hearts are shown, scale bar is 50 μm. **e** Masson’s staining of the hearts are shown, scale bar is 50 μm. **f** Echocardiographic analysis are shown. **g–k** Echocardiographic parameters are demonstrated. The left ventricular internal diameter (**g**), interventricular septum (**h**), left ventricular posterior wall (**i**), ejection fraction (**j**), cardiac output (**k**) and **l** heart weight versus body weight are measured (*n* = 6). **m** Hypertrophy markers β-MHC and fibrosis marker Col1a, are measured by western blotting analysis (*n* = 6)

On the other hand, to evaluate the influences of miR-455-5p overexpression in vivo, mice were administrated with NC agomir/miR-455-5p agomir by tail vain injection. The workflows of the procedures about the experiment are presented in Fig. S1b. The efficiency of miR-455-5p agomir in mice heart tissue were validated (Fig. S3d). As a result, compared to NC agomir group, cardiac morphology and the size of cardiomyocytes were bigger, hypertrophic and fibrotic responses were intenser in miR-455-5p agomir group (Fig. 3a–e). Meanwhile, LVPWd, IVSTd, HW/BW ratio were significantly elevated, whereas EF and CO were evidently declined in mice administrated with miR-455-5p agomir compared with their NC agomir group (Fig. 3f–l). At the molecular level, β-MHC and Col1a were more abundant in miR-455-5p agomir mice than their NC agomir counterparts (Fig. 3m).

In summary, miR-455-5p contributes to the developments of pathological cardiac remodeling and left ventricular dysfunction, whereas inhibition of miR-455-5p significantly blocks their developments.

### MiR-455-5p directly targets to PRMT1

**Fig. 4:**
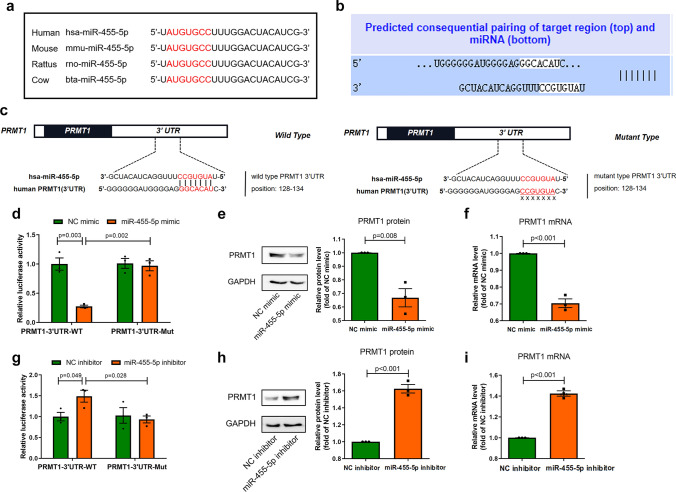
PRMT1 is the direct target of miR-455-5p. **a** Schematic representation of miR-455-5p sequence, especially of the binding sequence of miR-455-5p on 3’UTR of PRMT1 (in red), in mammals. **b** miRNA data base TargetScan showing the predicted target region on PRMT1 (top) and predicted binding region of miR-455-5p (bottom). **c** Schematic diagram representing the binding site of wild type (left) and mutant type (right) for miR-455-5p in the 3' UTR of PRMT1 in humans. **d** Luciferase assays showing that the binding capacity of miR-455-5p on 3’ UTR of PRMT1 in NRCMs transfected with miR-455-5p mimic (*n* = 3). **e**, **f** Western blotting and q-PCR assay showing PRMT1 protein (**e**) and mRNA levels (**f**) after treatment with miR-455-5p mimic at baseline (*n* = 3). **g** Luciferase assays showing that the binding capacity of miR-455-5p on 3’ UTR of PRMT1 in NRCMs transfected with miR-455-5p inhibitor (*n* = 3). **h-i** Western blotting and q-PCR assay showing PRMT1 protein (**h**) and mRNA levels (**i**) after treatment with miR-455-5p inhibitor at baseline (*n* = 3)

According to NCBI database, the base sequences of miR-455-5p in human, rat, mouse and cow are identical, indicating that miR-455-5p is highly conserved among mammals (Fig. 4a). Recent studies revealed that miRNAs regulate mRNA by binding to the 3’ untranslated region (UTR) of mRNA and promote its degradation [7, 31]. To find out the target mRNA, we used computational approaches to predict the targeted 3’ UTR of miR-455-5p. The TargetScan algorithm (http://www.targetscan.org) revealed a potential binding of miR-455-5p to PRMT1, which is a crucial enzyme that regulated asymmetric di-methylation of arginine residues in its substrates (Fig. 4b). Then, PRMT1 as a target of miR-455-5p was further validated by luciferase assays. The potential binding site of PRMT1 was mutated from *GGCACAT* to *CCGTGTA* via site-directed mutagenesis (Fig. 4c). Transfection of a plasmid containing the luciferase sequence followed by the native PRMT1 3’UTR, together with miR-455-5p mimic in NRCMs, gave rise to a 72% decrease in normalized luciferase activity. Furthermore, miR-455-5p barely bound to the mutant PRMT1 (Fig. 4d). In addition, PRMT1 mRNA and protein levels were significantly decreased in NRCMs transfected with miR-455-5p mimic (Fig. 4e–f). Similarly, PRMT1 protein level of heart tissue in miR-455-5p agomir group was lower than that in NC agomir group (Fig. S4). Thus, these results suggested that miR-455-5p suppresses the transcription of PRMT1. On the contrary, by transfection of a plasmid containing the luciferase sequence followed by the native PRMT1 3’ UTR, together with miR-455-5p inhibitor in NRCMs, we observed a significantly increase in normalized luciferase activity, but this result did not happen when native 3’ UTR was replaced by mutant 3’ UTR (Fig. 4g). Likewise, PRMT1 mRNA and protein levels were evidently upregulated in NRCMs transfected with miR-455-5p inhibitor (Fig. 4h, i).

Similar to PRMT1, PRMT2, PRMT3, PRMT4, PRMT6 and PRMT8 also regulate asymmetric di-methylation of arginine residues in their substrates. Since they functioned similarly and belonged to same subfamily of PRMTs, namely type I PRMTs. Therefore, it is necessary to investigate whether miR-455-5p also regulate the expressions of PRMT2, 3, 4, 6 and 8. As shown in Fig. S5a–b, both mRNA level and protein level of PRMT2, 3, 4, 6 and 8 were unchanged in NRCMs transfected with miR-455-5p mimic, indicating that miR-455-5p did not influence the expressions of PRMT2, 3, 4, 6 and 8.

In conclusion, PRMT1 is a direct target of miR-455-5p.

### PRMT1 and its methylation activity protect against cardiac hypertrophy and fibrosis

**Fig. 5:**
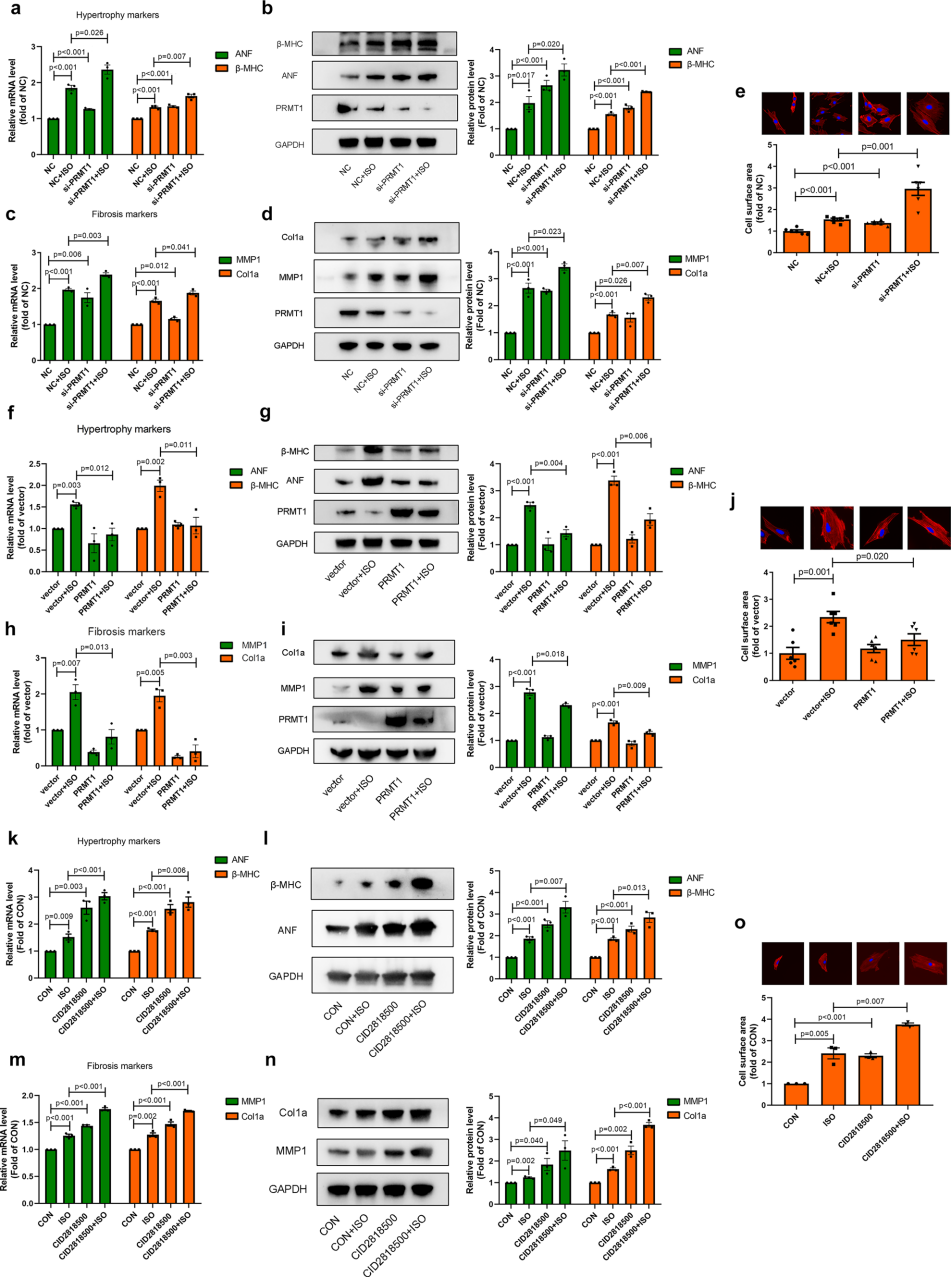
PRMT1 protects against cardiac hypertrophy and cardiac fibrosis. a-b mRNA levels (**a**) and protein levels (**b**) of ANF, β-MHC are measured in NRCMs transfected with NC or si-PRMT1 in the presence or absence of ISO (*n* = 3). **c, d** mRNA levels (**c**) and protein levels (**d**) of MMP1 and Col1a are measured in NRFBs transfected with NC or si-PRMT1 in the presence or absence of ISO (*n* = 3). **e** Cell surface areas are measured in NRCMs transfected with NC or si-PRMT1 in the presence or absence of ISO (*n* = 6). **f**, **g** mRNA levels (**f**) and protein levels (**g**) of ANF, β-MHC are measured in NRCMs transfected with vector or PRMT1 plasmid in the presence or absence of ISO (*n* = 3). **h**, **i** mRNA levels (**h**) and protein levels (**i**) of MMP1 and Col1a are measured in NRFBs transfected with vector or PRMT1 plasmid in the presence or absence of ISO (*n* = 3). **j** Cell surface area are measured in NRCMs transfected with vector or PRMT1 plasmid in the presence or absence of ISO (*n* = 6). **k, l** mRNA levels (**k**) and protein levels (**l**) of ANF, β-MHC are measured in NRCMs treated with 10 μM PRMT1 enzymatic inhibitor CID2818500 in the presence or absence of ISO (*n* = 3). **m–n** mRNA levels (**m**) and protein levels (**n**) of MMP1 and Col1a are measured in NRFBs treated with 10 μM PRMT1 enzymatic inhibitor CID2818500 in the presence or absence of ISO (*n* = 3). **o** Cell surface area are measured in NRCMs treated with 10 μM PRMT1 enzymatic inhibitor CID2818500 in the presence or absence of ISO (*n* = 3)

To determine the function of PRMT1 in cardiac hypertrophy and cardiac fibrosis, we performed loss-of-function and gain-of-function experiments by transfection with si-PRMT1 and PRMT1 overexpression plasmid. The efficiency of si-PRMT1 and PRMT1 overexpression plasmid were validated (Fig. S3e, f). Silencing of PRMT1 in NRCMs resulted in upregulation of hypertrophy markers (ANF and β-MHC) and fibrosis markers (Col1a and MMP1), as well as enlargement of surface area (Fig. 5a–e). Meanwhile, overexpression of PRMT1 suppressed ISO-induced upregulation of hypertrophy markers, fibrosis markers and enlargement of cell surface area (Fig. 5f–j). Besides, to investigate the function of methylation activity of PRMT1 in cardiac hypertrophy and cardiac fibrosis, a pharmacological inhibitor that specifically targeted to PRMT1, named CID2818500, was employed in NRCMs [32]. As a result, CID2818500 exacerbated ISO-induced hypertrophy and fibrotic responses, indicating that suppression of methylation activity of PRMT1 triggered cardiac hypertrophy and cardiac fibrosis (Fig. 5k–o).

Taken together, PRMT1 and its methylation protect against cardiac hypertrophy and fibrosis.

### PRMT1 activates the Notch signaling pathway by promoting asymmetric di-methylation on R1748, R1750, R1751 and R1752 of Notch1

**Fig. 6:**
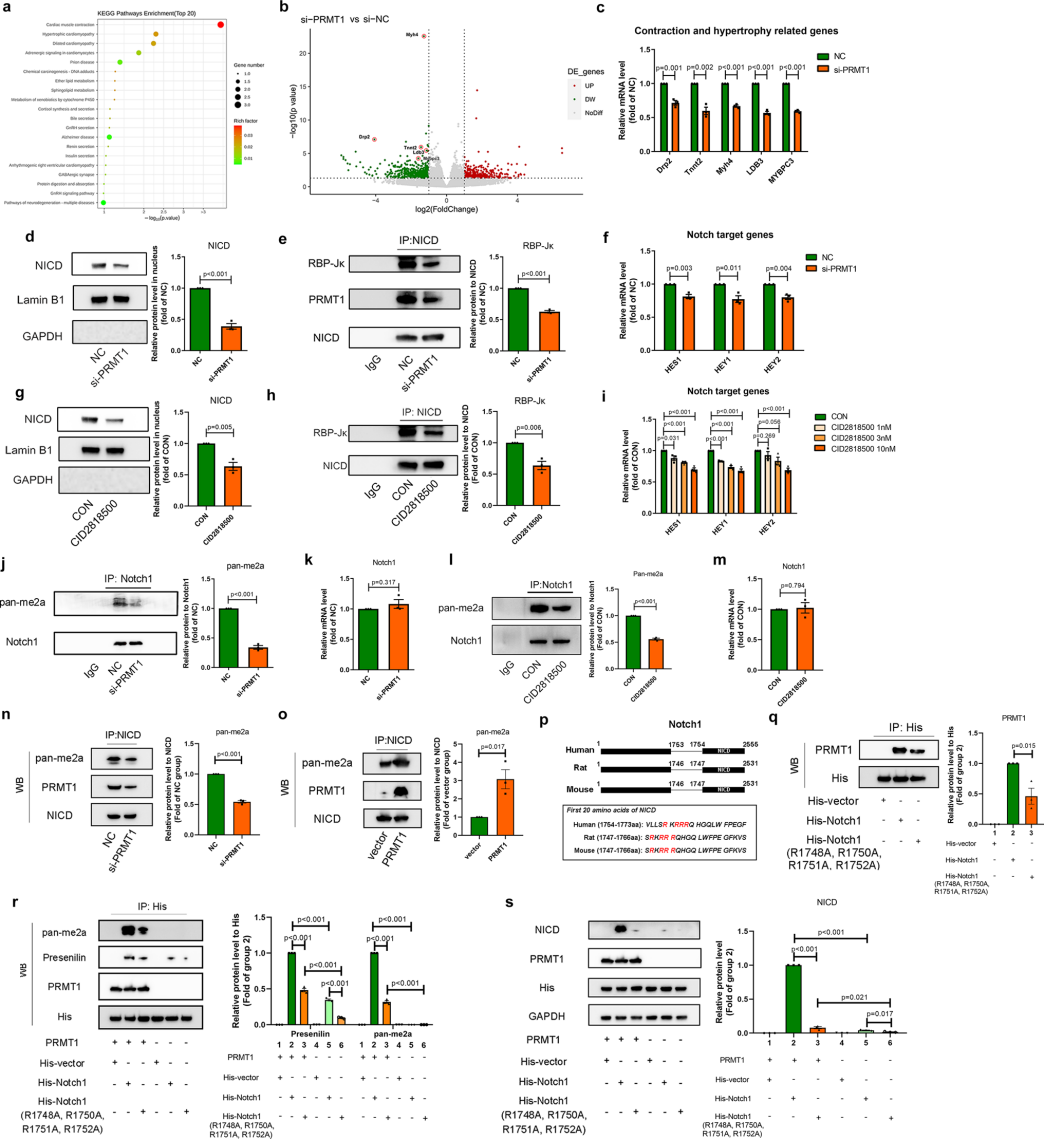
PRMT1 mediates asymmetric di-methylation of Notch1 and participates in activation of Notch signaling pathway. **a** KEGG analyzing the potential processes in NRCMs regulated by PRMT1. **b** mRNA sequencing comparing expression of mRNAs in si-PRMT1 vs si-NC transfected NRCMs is utilized to demonstrate target genes (*n* = 3). The cutoff fold change ≥ 1.5 and *p* value < 0.05 are utilized to identify differentially expressed genes. Non-changed genes are shown in grey color. Red color is indicative of up-regulated genes and green is indicative of down-regulated genes. **c** Q-PCR assay showing the level of contraction and hypertrophy related genes Drp2, Tnnt2, Myh4, LDB3 and MYBPC3 in NRCMs transfected with NC or si-PRMT1 (*n* = 3). **d, e** Western blotting showing nucleus protein level of NICD (**d**) and Co-IP assay showing the interaction between NICD and RBP-Jκ (**e**) in NRCMs transfected with NC or si-PRMT1 (*n* = 3). **f** Q-PCR assay showing the level of Notch target genes HES1, HEY1, HEY2 in NRCMs transfected with NC or si-PRMT1 (*n* = 3). **g, h** Western blotting showing nucleus protein level of NICD (**g**) and Co-IP assay showing the interaction between NICD and RBP-Jκ (**h**) in NRCMs treated with negative control solvent or PRMT1 enzymatic inhibitor CID2818500 (*n* = 3). **i** Q-PCR assay showing the level of Notch target genes HES1, HEY1, HEY2 in NRCMs treated with negative control solvent or PRMT1 enzymatic inhibitor CID2818500 at 1 nM, 3 nM or 10Nm (*n* = 3). **j** Co-IP assay showing asymmetric di-methylation level of Notch1 in NRCMs transfected with NC or si-PRMT1 (*n* = 3). **k** Q-PCR assay showing mRNA level in NRCMs transfected with NC or si-PRMT1 (*n* = 3). **l** Co-IP assay showing asymmetric di-methylation level of Notch1 in NRCMs treated with negative control solvent or PRMT1 enzymatic inhibitor CID2818500 (*n* = 3). **m** Q-PCR assay showing mRNA level in NRCMs treated with negative control solvent or PRMT1 enzymatic inhibitor CID2818500 (*n* = 3). **n, o** Co-IP assay showing the asymmetric di-methylation level of NICD and the affinity of NICD and PRMT1 in NRCMs transfected with si-PRMT1 (**n**) or PRMT1 plasmid (**o**) (*n* = 3). **p** Schematic diagram showing locations of NICD in Notch1 protein and the sequences of the first 20 amino acids of NICD (*n* = 3). **q** Co-IP assay showing the affinity of wild-type His-tag Notch1 or mutant-type His-tag Notch1 (R1748A, R1750A, R1751A, R1752A) and PRMT1 in HEK293A cells (*n* = 3). **r** Co-IP assay showing the influence of R1748, R1750, R1751, R1752 on the asymmetric di-methylation level of Notch1 protein and the affinity of Presenilin and Notch1 in NRCMs His-tag vector, wild-type His-tag Notch1 or mutant-type His-tag Notch1, in the presence or absence of PRMT1 (*n* = 3). **s** Western blotting assay showing the NICD level in HEK293A cells transfected with His-tag vector, wild-type His-tag Notch1 or mutant-type His-tag Notch1, in the presence or absence of PRMT1 (*n* = 3)

To obtain insights into the mechanism of PRMT1, we performed mRNA sequencing and screened the genes and processes involved in cardiac remodeling by transfection of si-PRMT1 or si-NC in NRCMs. The top 20 processes involved in PRMT1 silencing were presented in KEGG pathway enrichment plot (Fig. 6a). Of the top ranking processes, cardiac muscle contraction (Rank 1), hypertrophic cardiomyopathy (Rank 2), dilated cardiomyopathy (Rank 3) and adrenergic signaling (Rank 4) are known as critical processes in cardiac remodeling, which supported our previously mentioned hypothesis that PRMT1 regulates cardiac remodeling. Besides, according to the results demonstrated in volcano plot, several top ranking genes (e.g. Drp2, Tnnt2, Myh4 and LDB3) were relevant to the top ranking processes like cardiac muscle contraction and hypertrophic cardiomyopathy (Fig. 6b and Supplementary Table S6), further q-PCR assay confirmed that these top ranking genes were also downregulated by si-PRMT1, indicating the importance of PRMT1 in regulating cardiac muscle contraction and hypertrophic cardiomyopathy in pathological cardiac remodeling (Fig. 6c).

Then, to clarify the underlying signaling pathway regulated by PRMT1 in cardiac remodeling, we screened some potential signaling pathways that were relevant to cardiac contraction and hypertrophic cardiomyopathy, including p-GSK3β/GSK3β, p-ERK/ERK, p-AKT/AKT and NICD/Notch1 [33–36]. As a result, knockdown of PRMT1 resulted in the suppression of Notch signaling pathway, since the protein level of the intracellular domain of Notch (NICD) in nucleus, affinity of NICD and transcription factor RBP-Jκ and mRNA levels of Notch signaling pathway target genes (HES1, HEY1, HEY2) were declined (Fig. 6d–f). Similarly, inhibition of asymmetric di-methylation activity of PRMT1 by CID2818500 resulted in the suppression of Notch signaling pathway (Fig. 6g–i), indicating that asymmetric di-methylation activity mediated by PRMT1 participated in the regulation of Notch signaling pathway. Meanwhile, no fluctuations were observed on the protein level of p-GSK3β/GSK3β, p-ERK/ERK and p-AKT/AKT in NRCMs transfected with si-PRMT1 (Fig. S6a), suggesting that Notch signaling pathway was the downstream pathway of PRMT1 in cardiac remodeling.

A research team reported that Notch1 is located in heart [22]. Hence, we hypothesized that Notch1 was a substrate which could be asymmetrically di-methylated by PRMT1. As a result, when PRMT1 was knocked down by si-PRMT1 or was enzymatic inhibited by CID2818500, asymmetric di-methylation level (pan-me2a) of Notch1 was decreased, while mRNA level of Notch1 remained unchanged (Fig. 6j–m). In addition, given that other type I PRMTs (PRMT2, PRMT3, PRMT4, PRMT6 and PRMT8) are also able to mediate asymmetric di-methylation, it is necessary to rule out the possibility of other type I PRMTs in regulating asymmetric di-methylation of Notch1. Therefore, we conducted Co-IP assay to examine whether other type I PRMTs are able to combine with Notch1. As a result, no bindings were detected between Notch1 and type I PRMTs except PRMT1 (Fig. S5c). Moreover, q-PCR and western blotting assay confirmed that knockdown of PRMT1 did not affect the expressions of other PRMTs, suggesting the low probability of indirectly influences on asymmetric di-methylation level of Notch1 mediated by the fluctuations of other type I PRMTs (Fig. S5d–e). Thus, PRMT1 regulates Notch1 in a post-translational manner but does not affect the transcriptional process of Notch1.

According to the previous studies [37], NICD is the key domain to active Notch signaling pathway. To clarify whether NICD is regulated by PRMT1, we investigated the asymmetric di-methylation level of NICD and the affinity of NICD and PRMT1. As shown in Fig. 6n, o, the asymmetric di-methylation level of NICD was downregulated in NRCMs transfected with si-PRMT1, and was upregulated in NRCMs transfected with PRMT1 plasmid. Besides, NICD was successfully enriched by anti-PRMT1 antibody, indicating that NICD can be modified by PRMT1 in NRCMs. It is reported that NICD cleavage from Notch1 protein is the necessary process to activate Notch signaling pathway [38], to clarify the details of asymmetric di-methylation in NICD cleavage, we focused on the arginine residues of NICD which were close to the junction of NICD and the other part of Notch1. According to the NCBI protein database, NICD ranged from 1747 to 2531aa in Notch1. Among the arginine residues in NICD in mice, 1748aa, 1750aa, 1751aa, 1752aa (R1748, R1750, R1751, R1752) are very close to the junction of NICD and the other part of Notch1 (Fig. 6p). Thus, we hypothesized that R1748, R1750, R1751, R1752 are the crucial residues modified by PRMT1 and play important roles in the process of Notch1 cleavage and NICD releasing. To testify this hypothesis, we constructed a wild type Notch1 plasmid and a mutant Notch1 plasmid (R1748A, R1750A, R1751A, R1752A). As demonstrated in Fig. 6q, the affinity of mutant-type Notch1 and PRMT1 was significantly weaker than the affinity of wild-type Notch1 and PRMT1. Meanwhile, asymmetric di-methylation level of mutant-type Notch1 was much lower than that in mutant-type Notch1 (Fig. 6r). On the other hand, Presenilin is the catalytic subunit of γ-secretase, and γ-secretase is an intramembrane aspartyl protease that cleaves Notch1 within their transmembrane domains, resulting in NICD releasing [39, 40]. We examined the influence of R1748, R1750, R1751 and R1752 of Notch1 in recruiting Presenilin. As demonstrated in Fig. 6r, the affinity of Presenilin and wild-type Notch1 was significantly higher than the affinity of Presenilin and mutant-type Notch1, no matter in the presence or absence of PRMT1. The presence of PRMT1 also reinforced the affinity of Presenilin and Notch1 (both wild-type and mutant-type). Furthermore, NICD level was greatly reduced when wild-type Notch1 was replaced by mutant-type Notch1 or PRMT1 was absent in HEK293A cells (Fig. 6s). Thus, asymmetric di-methylation of R1748, R1750, R1751 and R1752 of Notch1 by PRMT1 facilitated the recruitment of Presenilin on Notch1, resulting in NICD releasing and activation of Notch signaling pathway.

In summary, the above observations suggest that PRMT1 activates the Notch signaling pathway by promoting asymmetric di-methylation on the arginine residues on R1748, R1750, R1751 and R1752 of Notch1.

### miR-455-5p blocks the Notch signaling pathway and induces pathological cardiac remodeling by targeting to PRMT1

**Fig. 7:**
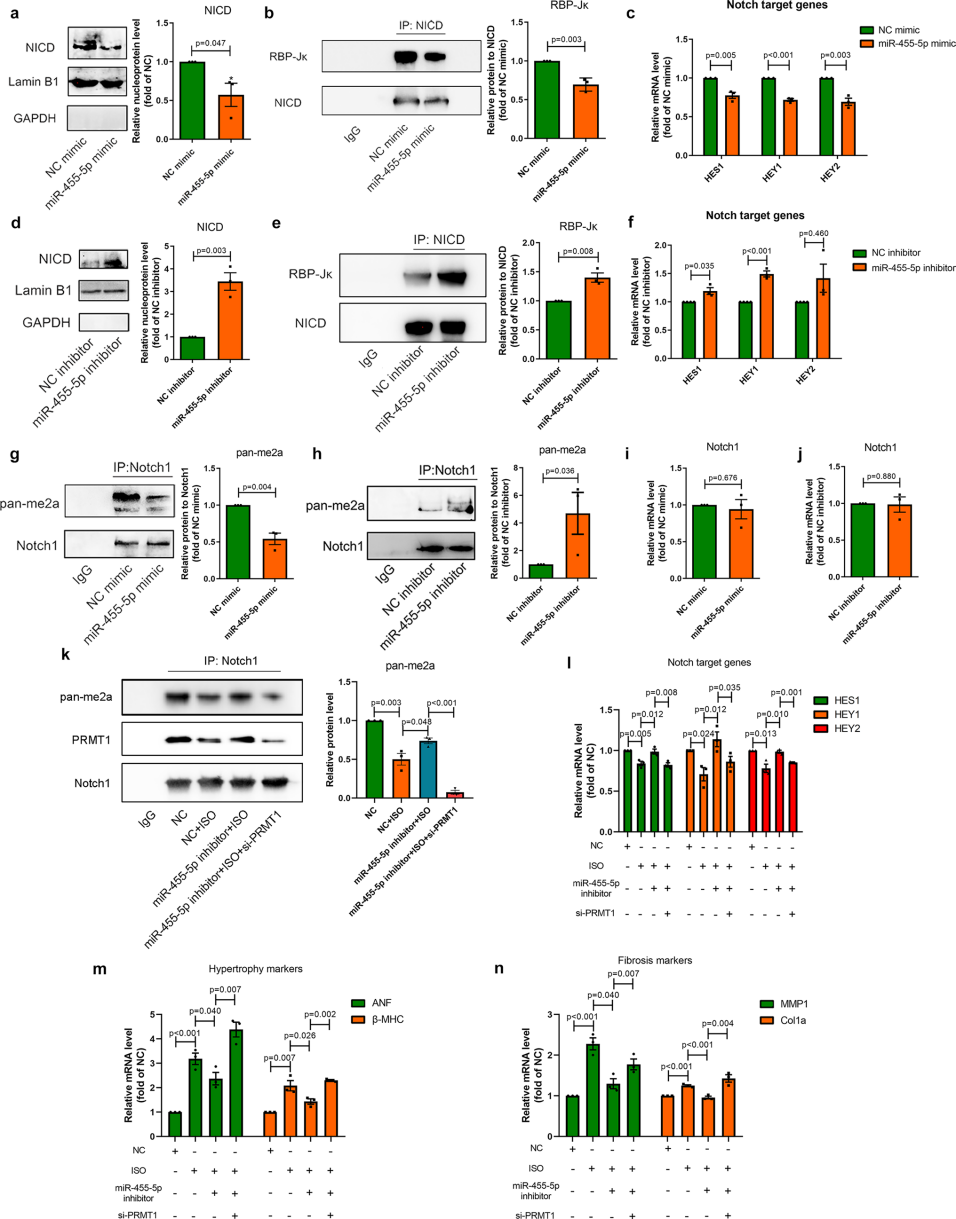
miR-455-5p provokes cardiac remodeling by inhibition of PRMT1-mediated Notch1 activation. **a** Western blotting showing the nuclear NICD protein level, **b** co-IP assay showing the interactions of NICD and RBP-Jκ, **c** q-PCR showing the mRNA level of Notch target genes in NRCMs transfected with NC mimic or miR-455-5p mimic (*n* = 3). **d** Western blotting showing the nuclear NICD protein level, **e** co-IP assay showing the interactions of NICD and RBP-Jκ, **f** q-PCR showing the mRNA level of Notch target genes in NRCMs transfected NC inhibitor or miR-455-5p inhibitor (*n* = 3). **g, h** Co-IP assay showing the asymmetric di-methylation of Notch1 in NRCMs under exposure of NC mimic/miR-455-5p mimic (**g**) or NC inhibitor/miR-455-5p inhibitor (**h**), IgG is regarded as negative control (*n* = 3). **i, j** Q-PCR showing PRMT1 mRNA level in NRCMs transfected with NC mimic/miR-455-5p mimic (**i**) or NC inhibitor/miR-455-5p inhibitor (**j**), *n* = 3. **k** Co-IP assay showing the asymmetric di-methylation of Notch1 in NRCMs transfected with or without miR-455-5p inhibitor or si-PRMT1, treated with or without ISO (*n* = 3). **l, m** Notch target genes HES1, HEY1, HEY2 (**l**), together with hypertrophy markers ANF and β-MHC (**m**) are measured in NRCMs transfected with or without miR-455-5p inhibitor or si-PRMT1, treated with or without ISO (*n* = 3). **n** Fibrosis markers MMP1 and Col1a are measured in NRFBs transfected with or without miR-455-5p inhibitor or si-PRMT1, treated with or without ISO (*n* = 3)

To investigate the regulatory role of miR-455-5p in Notch signaling pathway, NRCMs was transfected with miR-455-5p mimic and miR-455-5p inhibitor, respectively. Overexpression of miR-455-5p by miR-455-5p mimic obviously blunted the activation of Notch signaling pathway, as determined by decreasing protein level of NICD in nucleus, weaker interaction between RBP-Jκ and NICD and descending mRNA level of Notch target genes (Fig. 7a–c), whereas knockdown of miR-455-5p resulted in opposite responses (Fig. 7d–f). Besides, the levels of p-GSK3β/GSK3β, p-ERK/ERK and p-AKT/AKT remained unaffected in NRCMs transfected with miR-455-5p mimic, which were in line with the results in NRCMs transfected with si-PRMT1 (Fig. S6b). Thus, miR-455-5p is capable of suppressing Notch signaling pathway.

It is reported that Notch signaling pathway plays a key role in morphogenesis. During early period of heart development, myocardial Notch1 can guide cardiomyocytes to locate in the appropriate spatial position of the ventricular wall. Specific inhibition of cardiac Notch1 activity will lead to a decrease in ventricular volume and an increase in ventricular wall thickness [22, 41]. Similar to the results in these studies, we observed that miR-455-5p agomir-mediated overexpression of miR-455-5p induced ventricular wall thickening as presented by increased LVPWd and IVSTd value in vivo (Fig. 3h, i), whereas inhibition of miR-455-5p by antagomir impeded the increasing trend of LVPWd and IVSTd caused by ISO treatment (Fig. 2h, i). Additionally, some researchers reported that protein biogenesis, sarcomere organization, cardiac muscle contraction and oxidation of fatty acid alter abnormally in the development of ventricular wall thickening [42–46]. Not surprisingly, we also noticed that increasing levels of protein biogenesis-related gene S6K1, sarcomere-related genes Desmin and decreasing levels of muscle contraction-related gene MYBPC3 and fatty acid oxidation-related gene PPARα in NRCMs overexpressed miR-455-5p, while the opposite phenotypes appeared when miR-455-5p was knock-downed in NRCMs (Fig. S7a-b). Thus, the aforementioned results indicated that miR-455-5p plays an important role in ventricular wall thickening.

Next, we speculated that miR-455-5p may regulate ventricular wall thickening via Notch1 in pathological cardiac remodeling. Not surprisingly, asymmetric di-methylation level of Notch1 was obviously reduced in NRCMs treated with miR‐455-5p mimic, while significantly elevated in response to miR‐455-5p inhibitor (Fig. 7g, h). Besides, mRNA level of Notch1 was neither affected by miR‐455-5p mimic nor influenced by miR-455-5p inhibitor (Fig. 7i, j). Hence, these results suggest that miR-455-5p participates in the regulation of asymmetric di-methylation of Notch1, without affecting the transcription of Notch1.

The relationship of miR-455-5p and PRMT1 in the regulation of Notch signaling pathway was also investigated. It is interesting to note that reduced PRMT1 mRNA level was detected in NRCMs transfected with miR-455-5p mimic (Fig. 4f), whereas miR-455-5p level did not change in NRCMs transfected with si-PRMT1, indicating that the regulation pattern between miR-455-5p and PRMT1 was unidirectional, namely miR-455-5p-to-PRMT1 pattern (Fig. S8). Moreover, knockdown of PRMT1 by si-PRMT1 abrogated the increasing asymmetric di-methylation level of Notch1 mediated by miR-455-5p inhibitor (Fig. 7k). Silencing of PRMT1 impeded miR-455-5p inhibitor-mediated activation of Notch signaling pathway, reversed the anti-hypertrophic and anti-fibrotic effects of miR-455-5p inhibitor, as implied by the expressions of Notch target genes, hypertrophy markers and fibrosis markers (Fig. 7l–n). Therefore, we concluded that miR-455-5p functions as the upstream of PRMT1 in regulating Notch signaling pathway.

In conclusions, miR-455-5p blocks the Notch signaling pathway and induces pathological cardiac remodeling by targeting to PRMT1.

### Circulating miR-455-5p correlates with pathological cardiac remodeling in patients with hypertensive heart disease

**Fig. 8:**
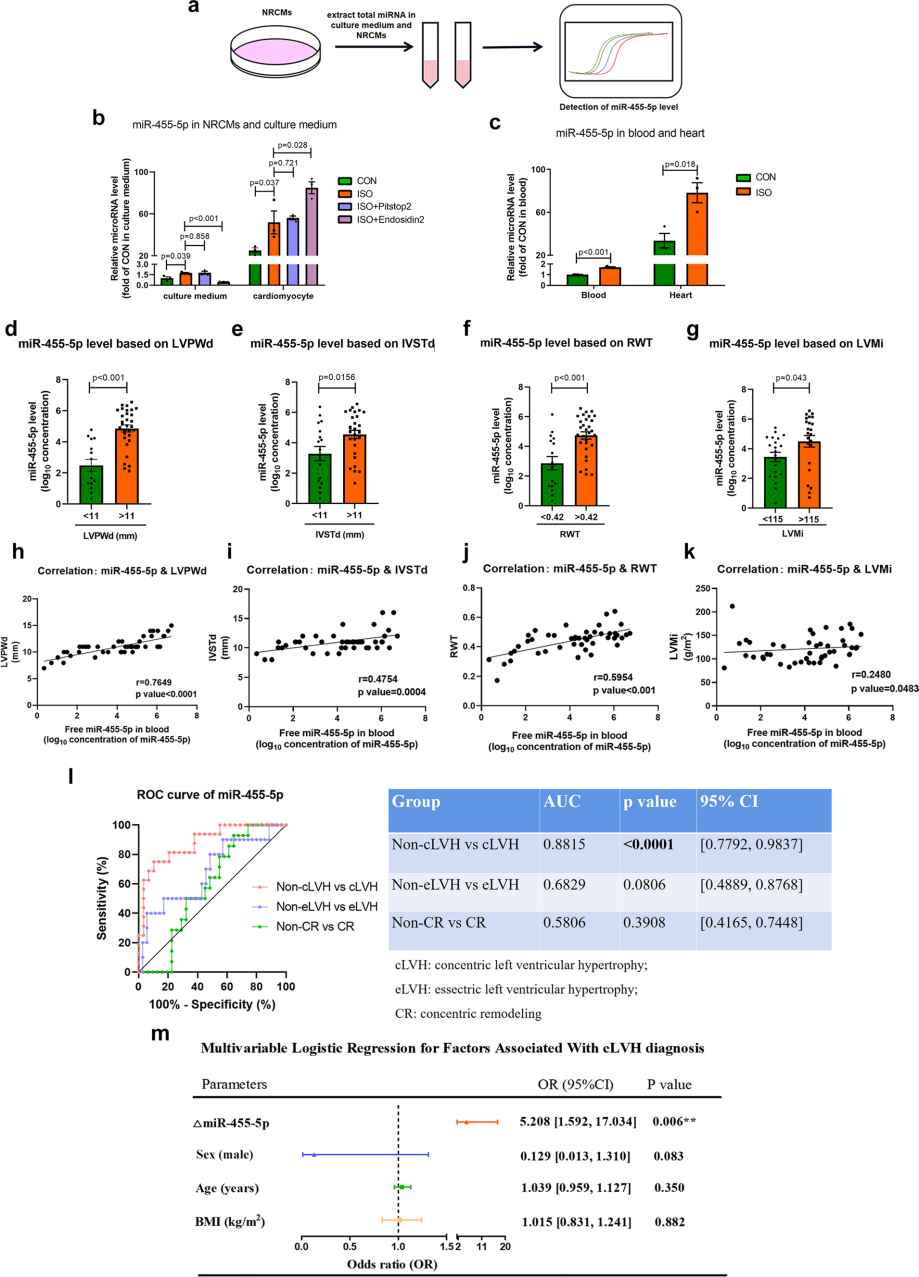
Investigation of circulating miR-455-5p in left ventricular cardiac remodeling in patients with hypertensive heart disease. **a** Extraction and measurement of total miRNA in culture medium and NRCMs are conducted as presented in the workflow. **b** q-PCR showing that miR-455-5p level in NRCMs/culture medium treated with only ISO, ISO + Pitstop2, ISO + Endosidin2 (*n* = 3). **c** Q-PCR showing that miR-455-5p level in heart tissue or blood treated with natural saline (CON group) or ISO (ISO group), *n* = 3; **d–g** Expression of circulating miR-455-5p is analyzed in 2 groups of patients based on LVPWd (**d**), IVSTd (**e**), RWT (**f**) and LVMi (**g**) by q-PCR assay, respectively. **h–k** Correlation analysis of miR-455-5p expression with the change in the level of LVPWd (**h**), IVSTd (**i**), RWT (**j**) and LVMi (**k**). Coefficient r value is calculated by Spearman coefficient method. *P* < 0.05 is considered as effective correlation (*n* = 46). **l** Receiver-operating characteristic curve (ROC) analysis for the prediction of cLVH/non-cLVH, CR/non-CR, eLVH/non-eLVH by using the miR-455-5p level. *P* < 0.05 is considered as reliable diagnostic indicator. **m** Forest plot demonstrated circulating △miR-455-5p (log_10_ concentration of miR-455-5p) as a crucial factor that associated with cLVH after adjustment by age, sex and BMI

It is reported that the origin of circulating miRNA depends on secretion of cardiomyocytes [47, 48]. Specifically, miRNA messages in cardiomyocytes are packaged into exosomes and then secreted to blood stream. Subsequently, these miRNAs regulate of gene expressions in recipient cells. To investigate whether miR-455-5p in heart can be secreted into blood stream, we measured the expression of miR-455-5p both in vitro and in vivo. Total miRNAs in NRCMs and culture medium was extracted and quantified according to workflow in Fig. 8a. Similar to the tendency of miR-455-5p in NRCMs treated with ISO, miR-455-5p level in culture medium also significantly elevated in ISO group. Besides, at baseline (CON group), the level of miR-455-5p in NRCMs was almost 20 fold more than that in culture medium, indicating that miR-455-5p was more abundant in NRCMs (Fig. 8b), this result was also supported by our observation that miR-455-5p level in heart tissue was much higher than that in blood stream (Fig. 8c). Based on these results, we assumed that miR-455-5p can be secreted into extracellular environment from cardiomyocytes. To validate this hypothesis, NRCMs were treated by Pitstops2 or Endosidin2, which was utilized to inhibit the process of endocytosis and secretion, respectively [49, 50]. As a result, inhibition of endocytosis by Pitstops2 treatment did not blunted ISO-mediated upregulation of miR-455-5p in NRCMs and culture medium. Meanwhile, inhibition of secretion by Endosidin2 significantly reduced miR-455-5p level in culture medium, as well as increasing miR-455-5p level in NRCMs (Fig. 8b). Therefore, the above observations suggest that miR-455-5p in heart can be secreted into blood stream.

Hypertension is a key factor in the development of pathological cardiac remodeling, thus, to identify circulating miRNAs in patients with pathological cardiac remodeling, we studied circulating miR-455-5p in patients with hypertensive heart disease. Briefly, 46 patients diagnosed with hypertensive heart disease were divided into lower group and higher group based on their cutoff LVPWd (≤ 11 mm or > 11 mm), IVSTd (≤ 11 mm or > 11 mm), RWT (≤ 0.42 or > 0.42) and LVMi (≤ 115 g/m^2^ or > 115 g/m^2^) as previously reported, respectively [51, 52]. As demonstrated in Fig. 8d–g, circulating miR-455-5p level was higher in patients with bigger LVPWd, IVSTd, RWT and LVMi. Furthermore, our data revealed that the circulating miR-455-5p level positively correlates with LVPWd, IVSTd, RWT and LVMi, indicating the predictive value of circulating miR-455-5p in pathological cardiac remodeling (Fig. 8h–k).

According to previously reported classification, hypertension-induced pathological cardiac remodeling is often classified into three different geometric patterns: concentric remodeling (CR), concentric left ventricular hypertrophy (cLVH), and eccentric left ventricular hypertrophy (eLVH) [53, 54]. Therefore, in order to explore the correlation of circulating miR-455-5p in different geometric patterns of pathological cardiac remodeling, we divided the cohort into four groups: patients with normal cardiac geometry, CR, cLVH and eLVH in patients with hypertensive heart diseases. Baseline characteristic demonstrated that there were no significant differences with regard to age (*P* = 0.479), sex (*P* = 0.252) and BMI (*P* = 0.094) between four groups. Besides, comorbidities that could influence the cardiac remodeling (e.g., hypertension, diabetes, coronary artery diseases and valvular heart diseases) were also similar among the groups. Of note, we observed that in patients with CR, cLVH or eLVH, the miR-455-5p level was robustly higher than that of patients in normal cardiac geometry group, with decreasing LVEF and increasing pro-BNP level, which suggests that the miR-455-5p level is associated with cardiac function and sensitive to different geometric patterns of pathological cardiac remodeling (Table 1).

**Table 1:** Baseline characteristics of the study population

Parameters	Total	Normal	cLVH	CR	eLVH	*P* value
Age (y)	62.3 ± 2.0	66.6 ± 7.1	64.8 ± 2.8	57.5 ± 4.1	63.0 ± 4.2	0.479
Male (*n*, total)	32 (46)	2 (5)	10 (16)	12 (14)	8 (11)	0.252
MiR-455-5p (ng/100 ml)	485.1 ± 153.1	3.9 ± 2.9	1186.0 ± 374.0	121.0 ± 74.7	147.1 ± 127.2	0.007
BMI (kg/m^2^)	25.7 ± 3.9	24.0 ± 2.1	26.1 ± 1.2	26.9 ± 0.7	24.7 ± 1.4	0.094
SBP (mmHg)	134.9 ± 3.7	123.6 ± 9.2	141.2 ± 7.5	140.1 ± 3.8	124.4 ± 8.0	0.198
DBP (mmHg)	82.9 ± 2.5	81.0 ± 6.3	78.6 ± 3.3	95.6 ± 4.3	73.7 ± 4.5	0.003
NT-proBNP (pg/ml)	3066 ± 1219	301 ± 233	4908 ± 2752	225 ± 101	4848 ± 2651	0.366
**Medical history**						
HT (*n*, total)	42 (46)	4 (5)	16 (16)	14 (14)	8 (11)	0.094
DM (*n*, total)	24 (46)	3 (5)	11 (16)	4 (14)	6 (11)	0.177
CAD (*n*, total)	41 (46)	4 (5)	15 (16)	11 (14)	11 (11)	0.308
VHD (*n*, total)	3 (46)	0 (5)	2 (16)	0 (14)	1 (11)	0.520
**Echocardiographic baseline**						
LVEF (%)	58.6 ± 2.16	66.3 ± 3.5	61.9 ± 1.1	66.2 ± 1.4	42.8 ± 5.4	< 0.001
LVIDd (mm)	51.0 ± 1.3	51.0 ± 2.1	48.7 ± 1.3	46.2 ± 0.6	60.6 ± 3.5	< 0.001
IVSTd (mm)	10.9 ± 0.2	9.4 ± 0.2	11.8 ± 0.4	10.9 ± 0.2	10.2 ± 0.4	0.007
LVPWd (mm)	11.1 ± 0.3	8.8 ± 0.6	12.5 ± 0.4	10.9 ± 0.2	10.1 ± 0.3	< 0.001
E/A	0.8 ± 0.1	0.9 ± 0.2	0.8 ± 0.1	0.8 ± 0.1	0.9 ± 0.2	0.759

Baseline demographic, medical historical, and echocardiographic parameters of the study population are shown. *P* values reflect the comparison among four different groups, *P* < 0.05 was regarded as significant difference

*SBP* systolic blood pressure, *DBP* diastolic blood pressure, *HT* hypertension, *DM* diabetes mellitus, *CAD* coronary artery diseases, *VHD* valvular heart diseases, *E/A* E peak versus A peak ratio, indicates diastolic function of left ventricular

Next, in order to assess the diagnostic and prognostic value of miR-455-5p in patients with CR, cLVH and eLVH, a receiver-operating characteristic (ROC) curve analysis was performed. An intriguing finding was that circulating miR-455-5p level was a reliable diagnostic indicator of cLVH [area under curve (AUC) = 0.8815, *P* < 0.0001], whereas circulating miR-455-5p failed to predict the occurrences of CR (AUC = 0.5806, *P* = 0.3908) and eLVH (AUC = 0.6829, *P* = 0.0806) (Fig. 8l). In addition, to further assess potential factors related to cLVH, univariable logistic regression analysis was employed (Supplementary Table S7). As a result, circulating miR-455-5p was an independent risk factor for cLVH (OR, 3.382 [95% CI 1.540–7.431]; *P* = 0.002), even after adjustment for sex, age and BMI by multivariable logistic regression analysis (OR, 5.208 [95% CI 1.592–17.034]; *P* = 0.006) (Fig. 8m). On the basis of the aforementioned data, we concluded that circulating miR-455-5p correlates with pathological cardiac remodeling, which could be utilized as a potential indicator to diagnose cLVH in hypertensive heart disease.

## Discussion

Pathological cardiac remodeling refers to a series of alterations including structural abnormalities, metabolic disorders and the inefficient pattern of energy utilization in failing myocardium, which eventually contribute to cardiac dysfunction. The major finding of our study was that miR-455-5p suppressed PRMT1 in failing myocardium and resulted in hypo-methylation level of Notch1. We demonstrated that miR-455-5p regulated myocardial PRMT1 expression via directly targeting at 3’UTR of the PRMT1 mRNA. This effect led to declining asymmetric di-methylation level on R1748, R1750, R1751 and R1752 of Notch1 and inactivation of Notch signaling pathway in failing myocardium, thus contributed to ventricular wall thickening that is associated with cardiac dysfunction.

According to previous studies, miR-455-5p plays a complex role in cardiovascular system. For example, some researchers found that miR-455-5p mimic accelerated the progression of atrial fibrillation in mice [11], supporting that miR-455-5p functions as a damaging role in heart. Meanwhile, other researchers proposed opposite opinions. They reported that miR-455-5p protected cardiomyocytes against oxidative stress [55]. Besides, some researchers showed that lower miR-455-5p level in vascular smooth muscle cells are relevant to atherosclerosis [56]. However, due to the lack of comprehensive data that was composed of the results of miR-455-5p in vitro, in vivo and at clinical aspects in these studies, it is not very easy to convince the researchers holding the opposite view. In this study, we investigated the function of miR-455-5p from different aspects by several methods. Via q-PCR assay, we detected miR-455-5p was more abundant in heart than spleen, lung, liver and kidney. In vitro, overexpression of miR-455-5p aggravated ISO-induced hypertrophy and fibrosis responses, while knockdown of miR-455-5p by miR-455-5p inhibitor resulted in the opposite side (Fig. 1). Similarly, by tail intravenous injection of miR-455-5p agomir or antagomir, miR-455-5p was identified as a factor to provoke pathological cardiac remodeling in vivo, as implied by the observations of morphological appearance, immunohistochemical staining, echocardiography data and molecular markers indicated cardiac hypertrophy and cardiac fibrosis (Figs. 2, 3). It thus hinted us that miR-455-5p played a pivotal role in inducing pathological cardiac remodeling in vivo, which was in line with the results in vitro. Bioinformatics analysis followed by in vitro luciferase assay in NRCMs validated that PRMT1 was a direct target of miR-455-5p (Fig. 4). Moreover, PRMT1 was suppressed in mice heart tissue when mice received miR-455-5p agomir administration (Fig. S4), while other type I PRMTs including PRMT2, 3, 4, 6 and 8 were not affected by miR-455-5p mimic, thus the above-mentioned results ruled out the possibility that miR-455-5p regulates other type I PRMTs (Fig. S5). By treating NRCMs with the PRMT1 enzymatic inhibitor CID2818500, PRMT1 siRNA and PRMT1 plasmid, we found that PRMT1 and its enzymatic activity protected against cardiac hypertrophy and cardiac fibrosis in vitro (Fig. 5), this result was consistent with the phenotypes previously reported in PRMT1-knockout mice [21]. By screening the key proteins of the potential signaling pathways that were relevant to cardiac remodeling, Notch signaling pathway was revealed to be the downstream of PRMT1, in which we also observed decreased asymmetric di-methylation level on R1748, R1750, R1751 and R1752 of Notch1 (Fig. 6 and S6). We also demonstrated that NRCMs transfected with miR-455-5p mimic exhibited decreased asymmetric di-methylation level of Notch1 and inactivation of Notch signaling pathway, which were in line with the results in NRCMs treated with si-PRMT1 or CID2818500 (Fig. 7, S6). Also, miR-455-5p was confirmed to be upstream of PRMT1 in regulating Notch signaling pathway based on the following results: 1. PRMT1 mRNA level was reduced in NRCMs transfected with miR-455-5p mimic, but miR-455-5p level was unchanged in NRCMs transfected with si-PRMT1 (Fig. S8); 2. Silencing of PRMT1 not only abrogated the elevation of asymmetric di-methylation level induced by miR-455-5p inhibitor, but also invalidated anti-hypertrophic and anti-fibrotic effects produced by miR-455-5p inhibitor. Besides, miR-455-5p inhibitor-mediated activation of Notch signaling pathway was also interrupted by si-PRMT1 (Fig. 7k–n). Finally, we found that miR-455-5p in cardiomyocytes was able to secrete into extracellular environment. And we demonstrated a newly identified role for circulating miR-455-5p associated with pathological cardiac remodeling in patients with hypertensive heart diseases. The expression of circulating miR-455-5p was positively correlated with ventricular wall thickening (higher LVPWd, IVSTd, RWT and LVMi) and was found to have an excellent predictive value for a kind of geometric pattern in pathological cardiac remodeling, namely concentric cardiac remodeling (Fig. 8).

The arginine methyltransferase PRMT1 plays a pivotal role in the maintenance of cardiac function. Several recent studies revealed PRMT1-knockout mice hearts demonstrated various structural alterations which were similar to morphological and functional characteristics in human cardiomyopathies [20, 21, 57]. Basically, the involvement of PRMT1 in cardiovascular diseases is dependent on its post-translational activity on substrates. For instance, mutation at R145 of cTnI was associated with hypertrophic cardiomyopathy due to the inhibition of PRMT1-mediated methylation on R146/R148 [58]. PRMT1 interacted with and methylated CaMKII at arginine residues 9 and 275, leading to its protection against hypertrophic responses. Consistently, the protective role of PRMT1 was replicated in this study (Fig. 5). Besides, both baseline mRNA and protein level of PRMT1 were much higher than the other type I PRMTs, indicating its dominant role in Type I PRMTs in NRCMs (Fig. S5f, g). On the other hand, we also confirmed that only PRMT1 but no other type I PRMTs (2, 3, 4, 6 and 8) participated in asymmetric di-methylation of Notch1 (Fig. 6, S5). To step further, we also explored the potential arginine residues modified by PRMT1. We found that asymmetric di-methylation of R1748, R1750, R1751 and R1752, the closest arginine residues to the starting amino acid residue of NICD (1747aa), not only greatly affected the whole asymmetric di-methylation level of Notch1, but also facilitated the recruitment of Presenilin on Notch1, Notch1 cleavage and subsequent NICD releasing (Fig. 6), indicating asymmetric di-methylation of R1748, R1750, R1751 and R1752 altered the activation process of Notch signaling pathway as the consequence. However, whether there are some synergistic effects or competitive effects among the four mentioned arginine residues still unknown. Besides, whether there are some other critical arginine residues suitable for asymmetric di-methylation have not been determined yet. Due to the lack of computer aided analysis and mass spectrometry, so far we are unable to answer these questions. Future studies are warranted to elucidate more arginine residues on Notch1, the asymmetric di-methylation status of which is important for their function in pathological cardiac remodeling, as well as clarifying interaction among arginine residues of Notch1 in activating of Notch signaling pathway.

In this study, Notch signaling pathway was chosen as the crucial downstream of miR-455-5p/PRMT1 axis due to the following reasons: (1) By screening the key proteins of cardiac remodeling-associated signaling pathways, Notch signaling pathway was proven to be regulated by both miR-455-5p and PRMT1 (Figs. 6, 7, S6). (2) Previous studies had reported that myocardial Notch1 can guide cardiomyocytes to locate in the appropriate spatial position of the ventricular wall. Specific inhibition of cardiac Notch1 activity will lead to a decrease in ventricular volume and an increase in ventricular wall thickness [22], indicating the important role of Notch1 in ventricular thickening. In this study, it is interesting to note that the most significant elevation was observed in LVPWd and IVSTd value in mice overexpressed miR-455-5p (Fig. 3h, i), whereas inhibition of miR-455-5p reduced LVPWd and IVSTd mediated by ISO treatment (Fig. 2h, i). (3) At the molecular level, a series of protein including S6K1, Desmin, Dystrophin, MYBPC3, PPARα, which were closely relevant to ventricular wall thickening, were verified to be regulated by miR-455-5p (Fig. S7). Taken together, it is reasonable to study the relationship of miR-455-5p and Notch1 in cardiac remodeling, since both of them were verified to be relevant to ventricular wall thickening.

**Fig. 9:**
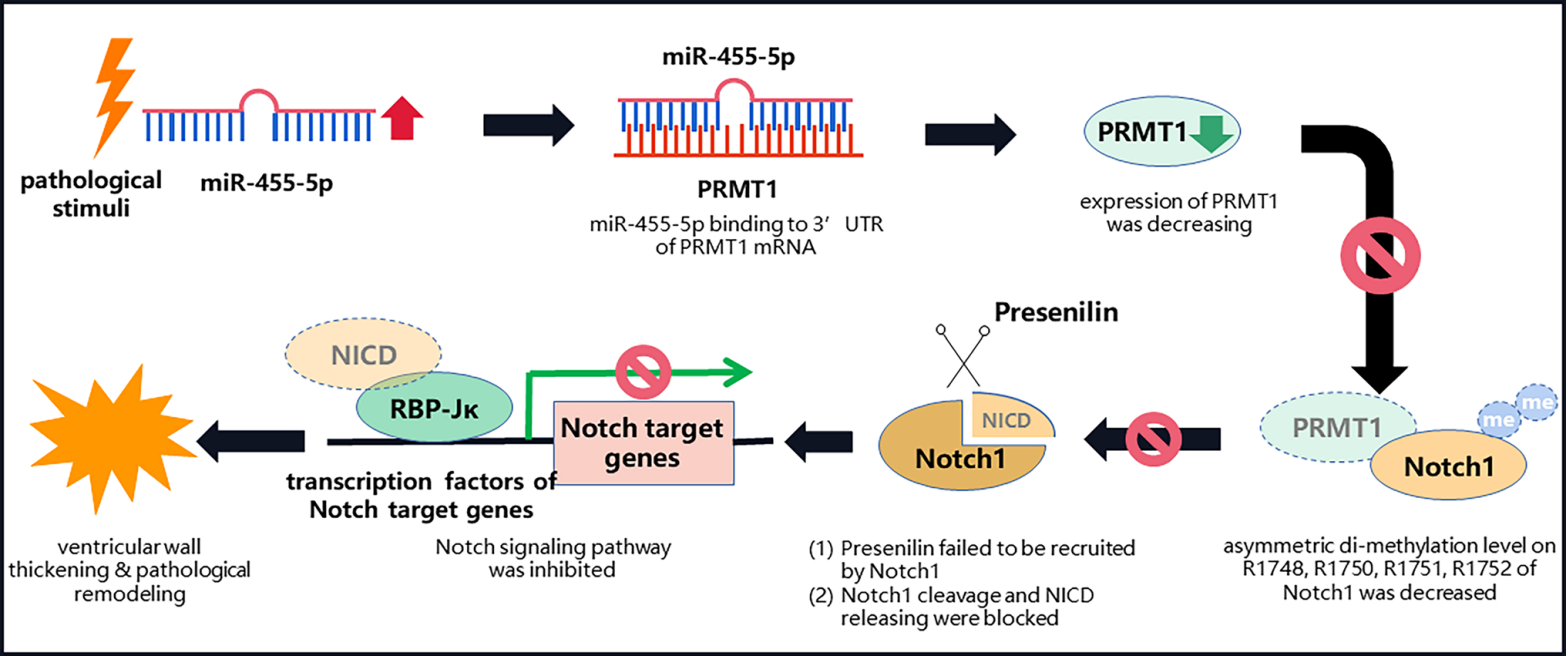
Graphical abstract demonstrates the mechanism of miR-455-5p in regulating pathological cardiac remodeling

To explore the clinical value of miR-455-5p in patients with cardiac remodeling, we chose hypertensive heart disease as our study object, because hypertension is a powerful factor to drive cardiac remodeling within the left ventricle, with strikingly high incidence worldwide [59, 60]. More importantly, hypertensive heart disease is a pressure-overload cardiac remodeling, which shares the same mechanism of our previously mentioned cardiac remodeling model in C57/BL6 mice induced by ISO [61, 62]. Based on this prerequisite, we noticed that circulating miR-455-5p level was not only higher in patients with higher LVPWd, IVSTd, RWT and LVMi, but was also positively correlated with the above 4 echocardiographic parameters. Besides, the expression tendency of circulating miR-455-5p was in line with our observation in ISO-induced hypertrophy model in vitro and in vivo (Fig. 8b, c), which supported our findings on NRCMs and mice. On the other hand, circulating miR-455-5p was validated to be effective in diagnosing cLVH (Fig. 8m), which was characterized by increased RWT and LVMi [63, 64]. Similarly, our study on mice administrated with miR-455-5p agomir, also demonstrated characteristics of cLVH, since increased IVSTd, LVPWd, HW/BW and unaffected LVIDd, LVEDV, LVESV were observed in mice of miR-455-5p agomir group (Fig. 3g–i, l, Supplementary Table S9). At the cellular level, cardiomyocytes in miR-455-5p mimic group was significantly wider than that in NC mimic group, while no difference in the length was detected between two groups (Fig. S9a). In addition, it has been reported that p90 ribosomal S6 kinase type 3 (RSK3) was a key protein in the development of concentric cardiac remodeling, overexpression of RSK3 increased width, without affecting the length of cardiomyocytes [65]. Actually, we observed a significant elevation of RSK3 mRNA level in cardiomyocytes transfected with miR-455-5p mimic (Fig. S9b). Therefore, the aforementioned similarities further confirmed our assumption that miR-455-5p play an important role in pathological cardiac remodeling, especially in cLVH (Fig. 9).

Taken together, we demonstrated a novel regulatory pathway in the failing myocardium that miR-455-5p suppresses PRMT1 and downregulates asymmetric di-methylation level of Notch1, leading to inactivation of Notch signaling pathway and promotion of ventricular wall thickening. Besides, we concluded that circulating miR-455-5p can be regarded as a diagnostic indicator in pathological cardiac remodeling, especially in the process of cLVH. These findings might provide novel targets for selective therapeutic interventions in the cardiovascular diseases related to pathological cardiac remodeling.

## Conclusions

In conclusion, the main finding of the present study is to clarify the function and mechanism of miR-455-5p in pathological cardiac remodeling. MiR-455-5p induces pathological cardiac remodeling by inhibition of PRMT1 transcription and subsequent inactivation of Notch1 activation. Circulating miR-455-5p is positively correlated with pathological cardiac remodeling and has a favorable diagnostic value of cLVH. Thus, inhibition of miR-455-5p or reactivation of PRMT1/Notch1 axis might serve as potential therapeutic strategies in treatment of pathological cardiac remodeling represented by ventricular wall thickening.

### Supplementary Information

Below is the link to the electronic supplementary material.

Supplementary file1 (SAV 8 KB)

Supplementary file2 (DOCX 23 KB)

Supplementary file3 (PDF 1589 KB)

Supplementary file4 (DOCX 28 KB)

Supplementary file5 (DOCX 24 KB)

Supplementary file6 (XLSX 634 KB)

## Data Availability

RNA-seq data generated in this study could be accessed in Supplementary file 6. Statistical data of patients without identifying information was available in Supplementary file 1.

## Abbreviations

AUC: Area under curve
BMI: Body mass index
cLVH: Concentric left ventricular hypertrophy
CR: Concentric remodeling
Drp2: Dystrophin-related protein 2
EF: Ejection fraction
eLVH: Eccentric left ventricular hypertrophy
HES1: HES family bHLH transcription factor 1
HEY1: Hairy/enhancer-of-split related with YRPW motif 1
HEY2: Hairy/enhancer-of-split related with YRPW motif 2
ISO: Isoprenaline
LDB3: LIM domain-binding protein 3
IVSTd: Interventricular septum on diastole stage
LVIDd: Left ventricular internal dimension on diastole stage
LVMi: Left ventricular mass index
LVPWd: Left ventricular posterior wall on diastole stage
MiRNA: MicroRNA
miR-455-5p: MicroRNA-455-5p
MYBPC3: Myosin-binding protein C3
Myh4: Myosin heavy chain 4
NICD: Intracellular domain of Notch
NRCM: Neonatal rat cardiomyocyte
NRFB: Neonatal rat cardiac fibroblasts
PRMT1: Protein arginine methyltransferase
ROC: Receiver-operating characteristic curve
RWT: Relative wall thickness
Tnnt2: Troponin T2, cardiac type
