# Supplementary material for: miR-455-5p promotes pathological cardiac remodeling via suppression of PRMT1-mediated Notch signaling pathway
Source: Cell Mol Life Sci. 2023 Nov 11;80(12):359. doi: 10.1007/s00018-023-04987-2 (PMC10640488; doi:10.1007/s00018-023-04987-2)
Supplement: Supplementary file 2 — Supplementary file2 (DOCX 23 KB) [file 18_2023_4987_MOESM2_ESM.docx]

**Supplementary Figure Legends**

miR-455-5p promotes pathological cardiac remodeling via suppression of PRMT1-mediated Notch signaling pathway

Sidong Cai^a,b^, Junlei Chang^b^, Mengqi Su^a^, Yinxia Wei^c^, Haoran Sun^d^, Cong Chen^*a^, Kai-Hang Yiu^*a^

^a^ Division of Cardiology, Department of Medicine, The University of Hong Kong-Shenzhen Hospital, Shenzhen, China;

^b^ Institute of Biomedicine and Biotechnology, Shenzhen Institute of Advanced Technology, Chinese Academy of Sciences, Shenzhen, China;

^c^ School of Public Health, Southern Medical University, Guangzhou, China;

^d^ Department of Clinical Microbiology and Infection Control, The University of Hong Kong-Shenzhen Hospital, Shenzhen, China.

* Correspondence to: Prof. Kai-Hang Yiu, email: khkyiu@hku.hk;

Prof. Cong Chen, email: chenc6@hku-szh.org.

**Fig. S1 Schemas illustrating the procedures of animial experiments.** (**a**) The miR-455-5p antagomir (20nmol/injection) or an equal dose of NC antagomir is injected via tail vain at day 1, 4, 8, 11, 15, 18, 22 and 25. At day 19-28, mice in NC antagomir+ISO and miR-455-5p antagomir group are subcutaneously administrated with ISO (1.5mg/kg/d), while the rest groups are subcutaneously administrated with equal dose of saline. At day 29, data about cardiac structure and function is collected by echocardiography. At day 30, all mice were sacrificed and the hearts are harvested for histological and molecular analysis. (**b**) The miR-455-5p agomir (10nmol/injection) or an equal dose of NC agomir is injected via tail vain at day 1, 4, 8, 11, 15, 18, 22 and 25. At day 29, data about cardiac structure and function is collected by echocardiography. At day 30, all mice are sacrificed and the hearts are harvested for histological and molecular analysis.

**Fig. S2 RNA purity and quantification are validated by Nanodrop One.** (**a**) RNA samples in this study are tested for purity and concentration by spectrophotometer Nanodrop One. OD260/OD280 between 1.8-2.0 and OD260/230 over 2.0 are acceptable for pure RNA. (**b**) RNA integrity and contamination test is conducted by denaturing agarose gel electrophoresis. RNA samples whose 28S and 18S bands are clear and sharp are considered as intact and uncontaminated.

**Fig. S3 Validation of the efficiency of miR-455-5p mimic/inhibitor, si-PRMT1 and PRMT1 plasmid in vitro, as well as miR-455-5p agomir/antagomir in vivo. a-b** NC inhibitor/miR-455-5p inhibitor (**a**) or NC mimic/miR-455-5p mimic (**b**) are transfected into cardiomyocytes in 6-well dishes for 48h, cardiomyocytes are harvested and miR-455-5p level are measured by q-PCR assay (n=3). **c-d** C57BL/6 mice are tail intravenously administrated with NC antgomir/miR-455-5p antgomir (**c**) or NC agomir/miR-455-5p agomir (**d**) for 4 weeks. Heart tissue are harvested and miR-455-5p level are examined by q-PCR assay (n=6). **e-f** NC/si-PRMT1 (**e**) or vector/PRMT1 plasmid (**f**) are transfected into cardiomyocytes in 6-well dishes for 48h, cardiomyocytes are harvested and PRMT1 level are measured by q-PCR assay (n=3).

**Fig. S4 Overexpression of miR-455-5p by agomir in vivo reduced protein level of PRMT1.** Protein of the heart tissue from C57BL/6 mice in NC agomir group and miR-455-5p agomir group is determined by western blotting (n=6).

**Fig. S5 miR-455-5p doesn’t affect the expressions of other type I PRMTs and Notch1 doesn’t bind to other type I PRMTs. a-b** q-PCR and western blotting showing mRNA levels (**a**) and protein levels (**b**) of PRMT2, PRMT3, PRMT4, PRMT6 and PRMT8 in NRCMs transfected with NC mimic or miR-455-5p mimic (n=3). **c** Co-IP assay showing the affinity of Notch1 with PRMT1, PRMT2, PRMT3, PRMT4, PRMT6 and PRMT8. IgG is considered as negative control. **d-e** q-PCR and western blotting showing mRNA levels (**d**) and protein levels (**e**) of PRMT1, PRMT2, PRMT3, PRMT4, PRMT6 and PRMT8 in NRCMs transfected with NC or si-PRMT1 (n=3). **f-g** q-PCR and western blotting showing baseline mRNA levels (**f**) and protein levels (**g**) of PRMT1, PRMT2, PRMT3, PRMT4, PRMT6 and PRMT8 in NRCMs (n=3).

**Fig. S6 Potential mechanisms that correlate with ventricular wall thickening are screened in NRCMs transfected with si-PRMT1 or miR-455-5p mimic. a** Western blotting showing the protein levels of p-GSK3β/GSK3β, p-ERK/ERK and p-AKT/AKT in NRCMs transfected with NC or si-PRMT1 (n=3). **b** Western blotting showing the protein levels of p-GSK3β/GSK3β, p-ERK/ERK and p-AKT/AKT in NRCMs transfected with NC mimic or miR-455-5p mimic (n=3).

**Fig. S7 miR-455-5p promotes protein biogenesis and sarcomere formation, as well as impairing cardiac muscle contraction and fatty acid oxidation capacity. a** q-PCR showing the mRNA levels of S6K1, Desmin, MYBPC3 and PPARα in NRCMs transfected with NC mimic or miR-455-5p mimic (n=3). **b** q-PCR showing the mRNA levels of S6K1, Desmin, MYBPC3 and PPARα in NRCMs transfected with NC inhibitor or miR-455-5p inhibitor (n=3). Abbreviation: S6K1, ribosomal protein S6 kinase 1; MYBPC3, myosin-binding protein C3; PPARα, peroxisome proliferator-activated receptor α.

**Fig. S8 miR-455-5p is unaffected by si-PRMT1.** Q-PCR assay showing the miR-455-5p level in NRCMs transfected with NC or si-PRMT1 (n=3).

**Fig. S9 miR-455-5p preferably increases the width rather length in concentric cardiac remodeling.** (**a**) The width and length are measured by rhodamine-phalloidin staining in NRCMs transfected with NC mimic or miR-455-5p mimic (n=3). (**b**) q-PCR assay showing the mRNA level of RSK3 in NRCMs transfected with NC mimic or miR-455-5p mimic (n=3). Abbreviation: RSK3, p90 ribosomal S6 kinase type 3.

**Fig. S10 PRMT1 exerts a stronger upregulation effect on NICD in NRCMs.** The NICD protein levels are measured by western blotting in NRCMs transfected with vector, PRMT1 plasmid, PRMT1 and PRMT4 plasmid, respectively (n=3).
