# Supplementary material for: miR-455-5p promotes pathological cardiac remodeling via suppression of PRMT1-mediated Notch signaling pathway
Source: Cell Mol Life Sci. 2023 Nov 11;80(12):359. doi: 10.1007/s00018-023-04987-2 (PMC10640488; doi:10.1007/s00018-023-04987-2)
Supplement: Supplementary file 3 — Supplementary file3 (PDF 1589 KB) [file 18_2023_4987_MOESM3_ESM.pdf]

Supplementary Figures

miR-455-5p promotes pathological cardiac remodeling via suppression of PRMT1-mediated Notch signaling pathway

Sidong Cai[a,b], Junlei Chang[b], Mengqi Su[a], Yinxia Wei[c], Haoran Sun[d], Cong Chen[*a], Kai-Hang Yiu[*a]

[a] Division of Cardiology, Department of Medicine, The University of Hong Kong-Shenzhen Hospital, Shenzhen, China;

[b] Institute of Biomedicine and Biotechnology, Shenzhen Institute of Advanced Technology, Chinese Academy of Sciences, Shenzhen, China;

[c] School of Public Health, Southern Medical University, Guangzhou, China;

[d] Department of Clinical Microbiology and Infection Control, The University of Hong Kong-Shenzhen Hospital, Shenzhen, China.

* Correspondence to: Prof. Kai-Hang Yiu, email: khkyiu@hku.hk;

Prof. Cong Chen, email: chenc6@hku-szh.org.

Fig. S1

a

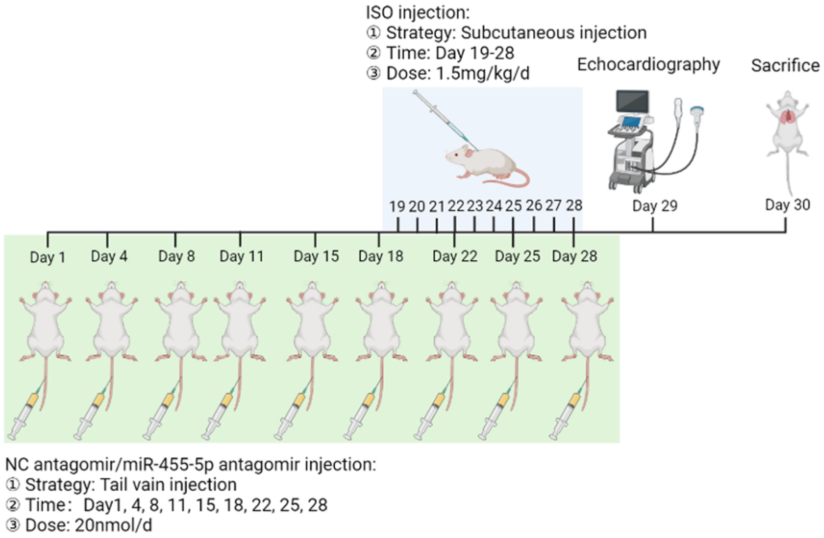
ISO injection:
① Strategy: Subcutaneous injection
② Time: Day 19-28
③ Dose: 1.5mg/kg/d
Echocardiography
Sacrifice
19 20 21 22 23 24 25 26 27 28
Day 29
Day 30
Day 1
Day 4
Day 8
Day 11
Day 15
Day 18
Day 22
Day 25
Day 28
NC antagomir/miR-455-5p antagomir injection:
① Strategy: Tail vain injection
② Time: Day1, 4, 8, 11, 15, 18, 22, 25, 28
③ Dose: 20nmol/d

b

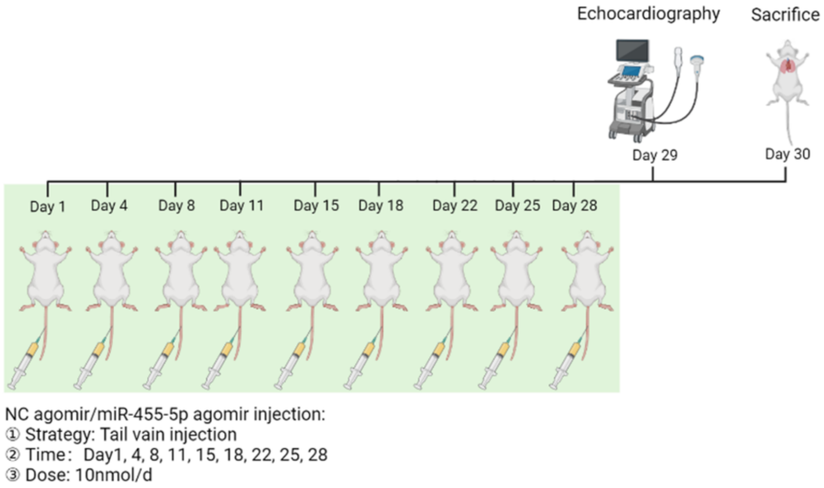
Echocardiography
Sacrifice
Day 29
Day 30
Day 1
Day 4
Day 8
Day 11
Day 15
Day 18
Day 22
Day 25
Day 28
NC agomir/miR-455-5p agomir injection:
① Strategy: Tail vain injection
② Time: Day1, 4, 8, 11, 15, 18, 22, 25, 28
③ Dose: 10nmol/d

Fig. S2

a

RNA quantification and quality assurance by Nanodrop One

Sample name	OD260/280*	OD260/230*	Concentration (ng/μl)	Volume (μl)	Quantity (ng)
NC mimic	1.97	2.05	823.15	30	24694.50
NC mimic+ISO	1.98	2.07	862.72	30	25881.60
miR-455-5p mimic	1.93	2.01	872.13	30	26163.90
MiR-455-5p+ISO	1.96	2.01	880.29	30	26408.70
NC inhibitor	1.92	2.05	795.10	30	23853.00
NC inhibitor+ISO	1.96	2.03	772.16	30	23164.80
miR-455-5p inhibitor	1.97	2.04	765.37	30	22961.10
miR-455-5p inhibitor+ISO	1.97	2.07	783.69	30	23510.70
CON	1.94	2.01	803.24	30	24097.20
ISO	1.96	2.02	807.39	30	24221.70
CID2818500	1.92	2.02	821.67	30	24650.10
CID2818500+ISO	1.97	2.04	833.17	30	24995.10
NC	1.95	2.06	892.79	30	26783.70
si-PRMT1	1.95	2.07	885.26	30	26557.80

* For spectrophotometer, OD260/280 between 1.8-2.0 and OD260/230 over 2.0 are acceptable for pure RNA.

b

RNA integrity and contamination test

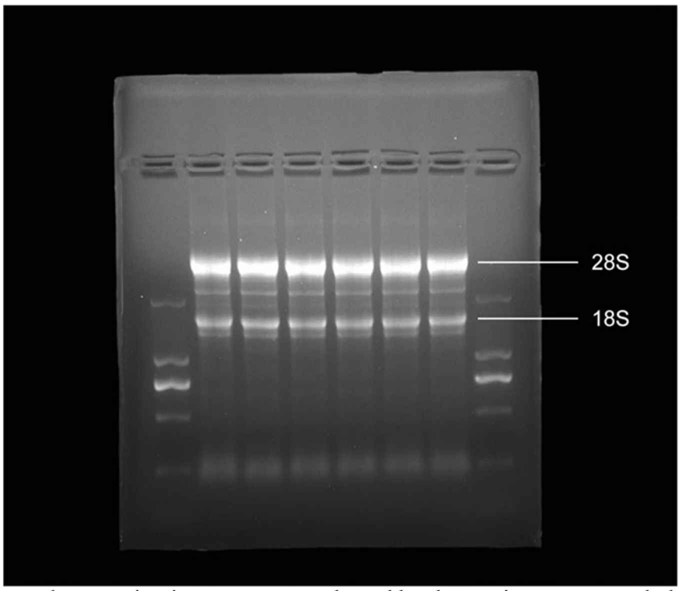

* RNA integrity and contamination test was conducted by denaturing agarose gel electrophoresis.

Fig. S3

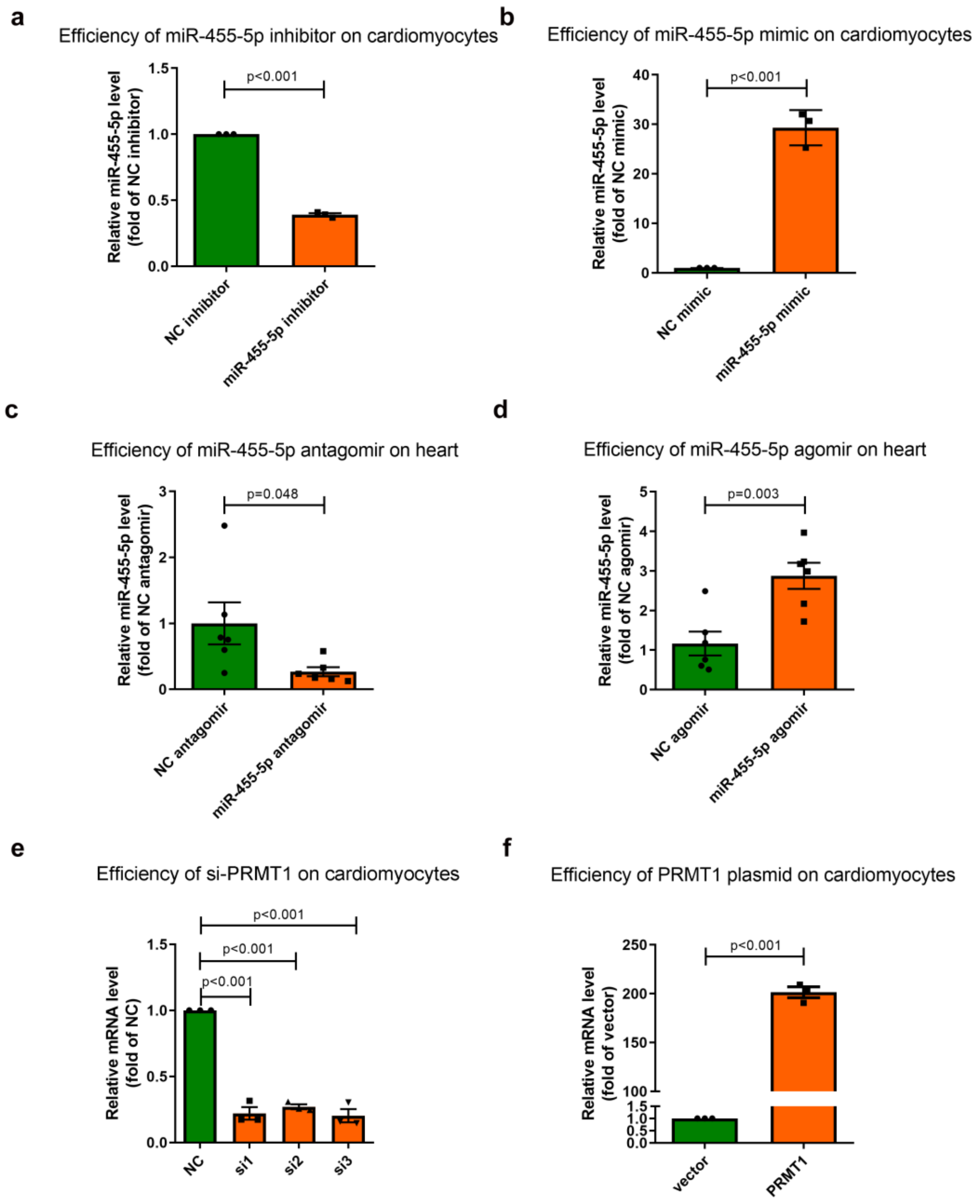
a
Efficiency of miR-455-5p inhibitor on cardiomyocytes
Relative miR-455-5p level (fold of NC inhibitor)
1.5
1.0
0.5
0.0
p<0.001
NC inhibitor
miR-455-5p inhibitor
b
Efficiency of miR-455-5p mimic on cardiomyocytes
Relative miR-455-5p level (fold of NC mimic)
40
30
20
10
0
p<0.001
NC mimic
miR-455-5p mimic
c
Efficiency of miR-455-5p antagomir on heart
Relative miR-455-5p level (fold of NC antagomir)
3
2
1
0
p=0.048
NC antagomir
miR-455-5p antagomir
d
Efficiency of miR-455-5p agomir on heart
Relative miR-455-5p level (fold of NC agomir)
5
4
3
2
1
0
p=0.003
NC agomir
miR-455-5p agomir
e
Efficiency of si-PRMT1 on cardiomyocytes
Relative mRNA level (fold of NC)
1.5
1.0
0.5
0.0
p<0.001
p<0.001
p<0.001
NC
si1
si2
si3
f
Efficiency of PRMT1 plasmid on cardiomyocytes
Relative mRNA level (fold of vector)
250
200
150
100
1.5
1.0
0.5
0.0
p<0.001
vector
PRMT1

Fig. S4

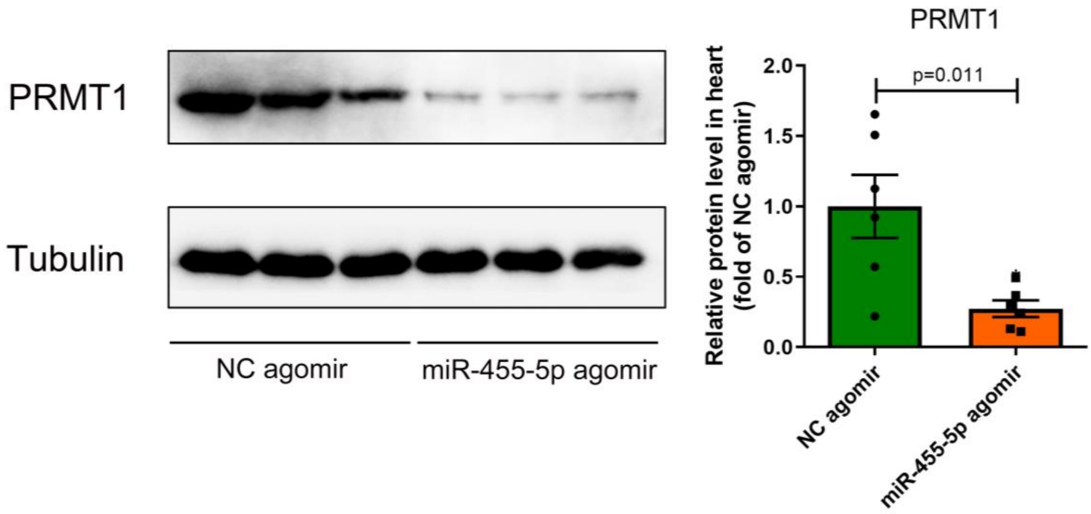
PRMT1
Tubulin
NC agomir
miR-455-5p agomir
PRMT1
p=0.011
Relative protein level in heart
(fold of NC agomir)
2.0
1.5
1.0
0.5
0.0
NC agomir
miR-455-5p agomir

Fig. S5

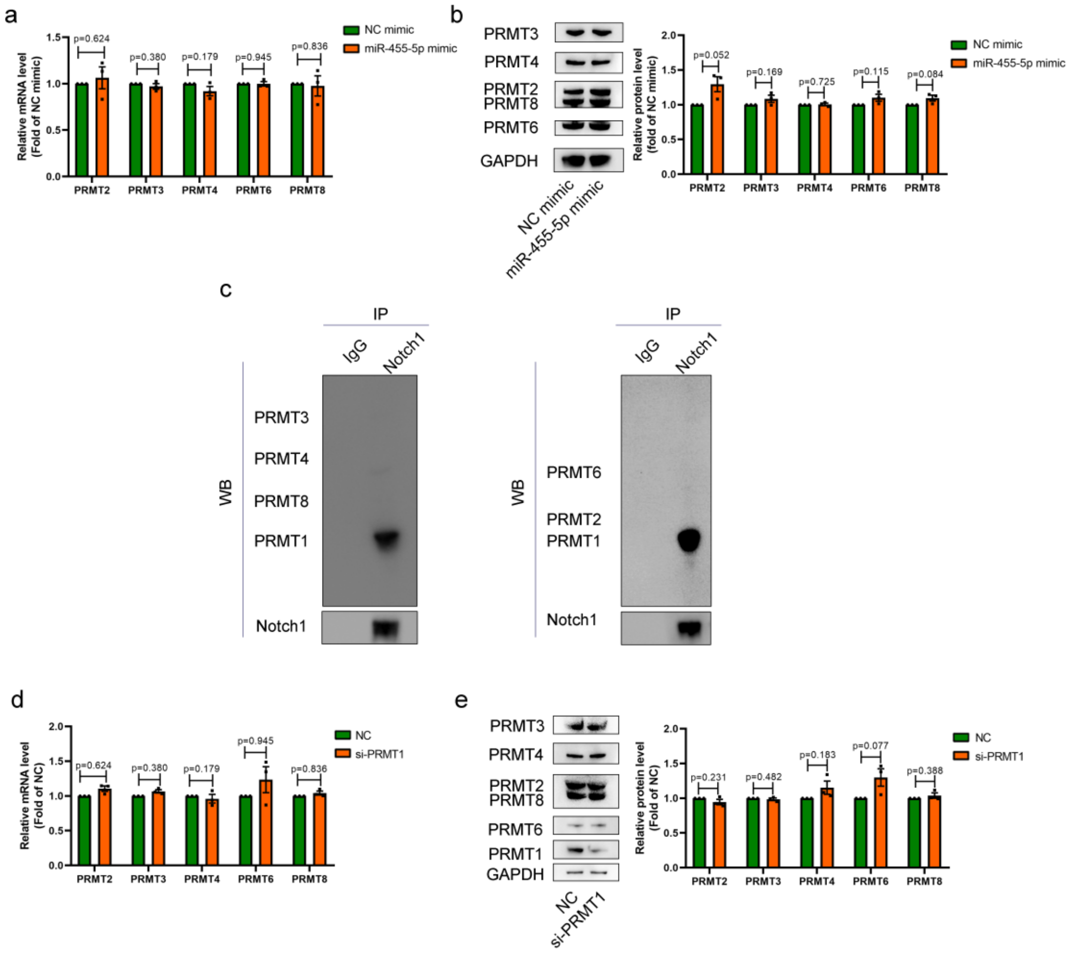

a
Relative mRNA level (Fold of NC mimic)
p=0.624
p=0.380
p=0.179
p=0.945
p=0.836
PRMT2
PRMT3
PRMT4
PRMT6
PRMT8
NC mimic
miR-455-5p mimic
b
PRMT3
PRMT4
PRMT2
PRMT8
PRMT6
GAPDH
NC mimic
miR-455-5p mimic
Relative protein level (fold of NC mimic)
p=0.052
p=0.169
p=0.725
p=0.115
p=0.084
c
IP
IgG
Notch1
WB
PRMT3
PRMT4
PRMT8
PRMT1
Notch1
PRMT6
PRMT2
PRMT1
d
Relative mRNA level (Fold of NC)
NC
si-PRMT1
e
PRMT1
GAPDH
Relative protein level (Fold of NC)
p=0.231
p=0.482
p=0.183
p=0.077
p=0.388

Fig. S6

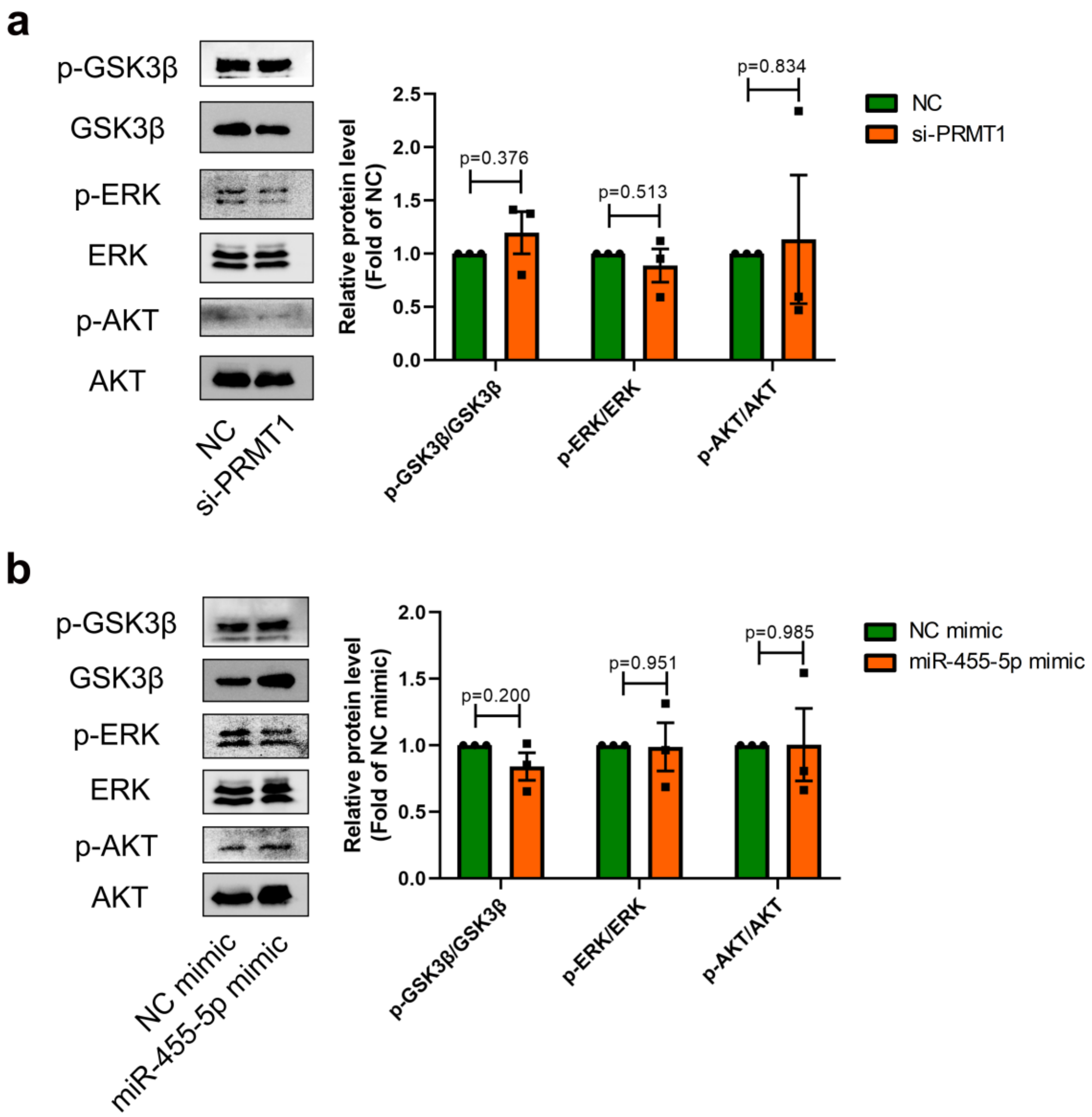
a
p-GSK3β
GSK3β
p-ERK
ERK
p-AKT
AKT
NC
si-PRMT1
Relative protein level
(Fold of NC)
2.5
2.0
1.5
1.0
0.5
0.0
p=0.376
p=0.513
p=0.834
p-GSK3β/GSK3β
p-ERK/ERK
p-AKT/AKT
NC
si-PRMT1
b
p-GSK3β
GSK3β
p-ERK
ERK
p-AKT
AKT
NC mimic
miR-455-5p mimic
Relative protein level
(Fold of NC mimic)
2.0
1.5
1.0
0.5
0.0
p=0.200
p=0.951
p=0.985
p-GSK3β/GSK3β
p-ERK/ERK
p-AKT/AKT
NC mimic
miR-455-5p mimic

Fig. S7

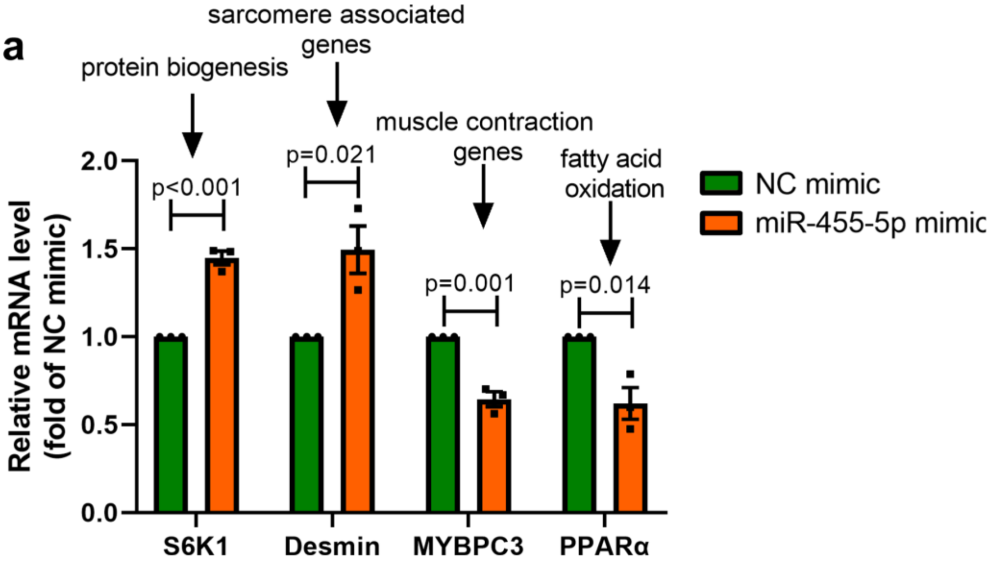
a
protein biogenesis
sarcomere associated genes
muscle contraction genes
fatty acid oxidation
p<0.001
p=0.021
p=0.001
p=0.014
Relative mRNA level (fold of NC mimic)
2.0
1.5
1.0
0.5
0.0
S6K1
Desmin
MYBPC3
PPARα
NC mimic
miR-455-5p mimic

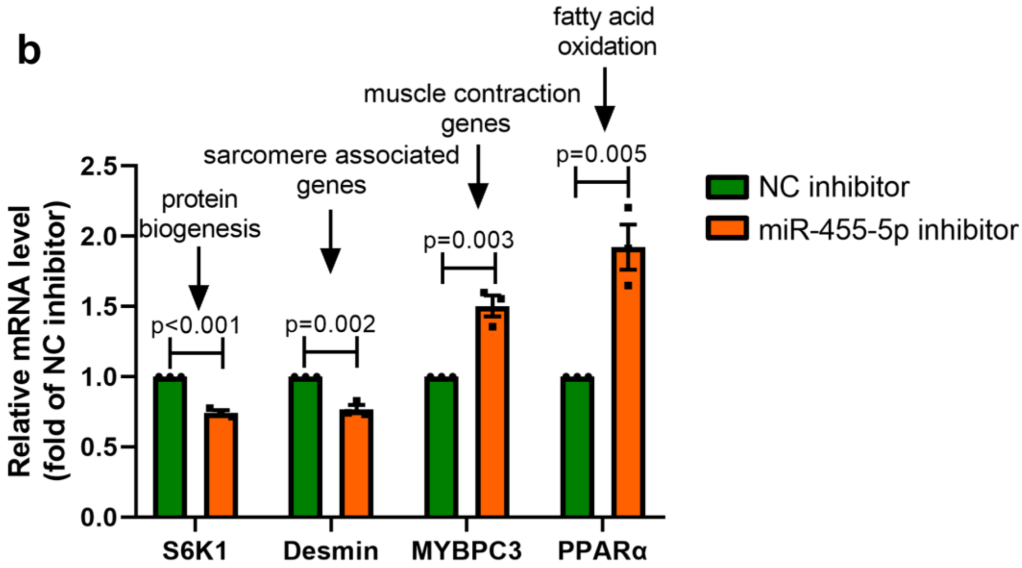
b
protein biogenesis
sarcomere associated genes
muscle contraction genes
fatty acid oxidation
p<0.001
p=0.002
p=0.003
p=0.005
Relative mRNA level (fold of NC inhibitor)
2.5
2.0
1.5
1.0
0.5
0.0
S6K1
Desmin
MYBPC3
PPARα
NC inhibitor
miR-455-5p inhibitor

Fig. S8

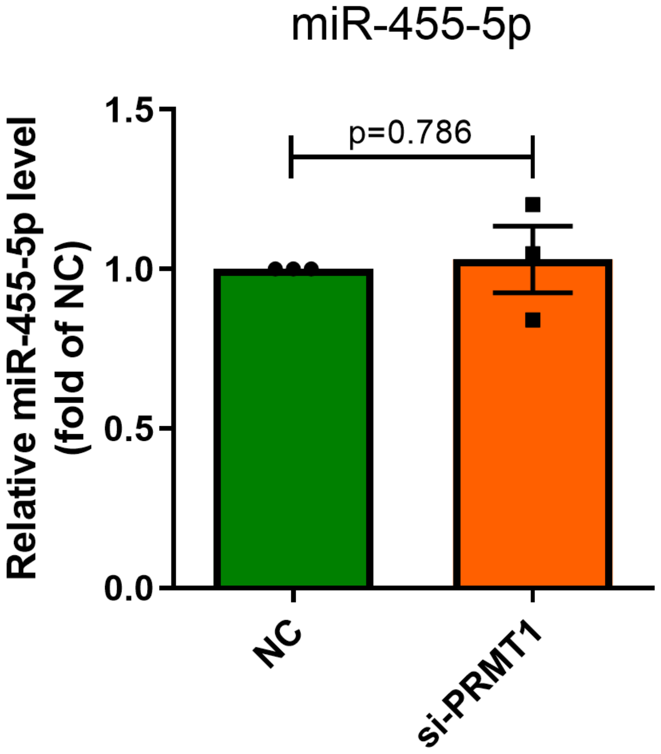

miR-455-5p
Relative miR-455-5p level
(fold of NC)
1.5
1.0
0.5
0.0
p=0.786
NC
si-PRMT1

Fig. S9

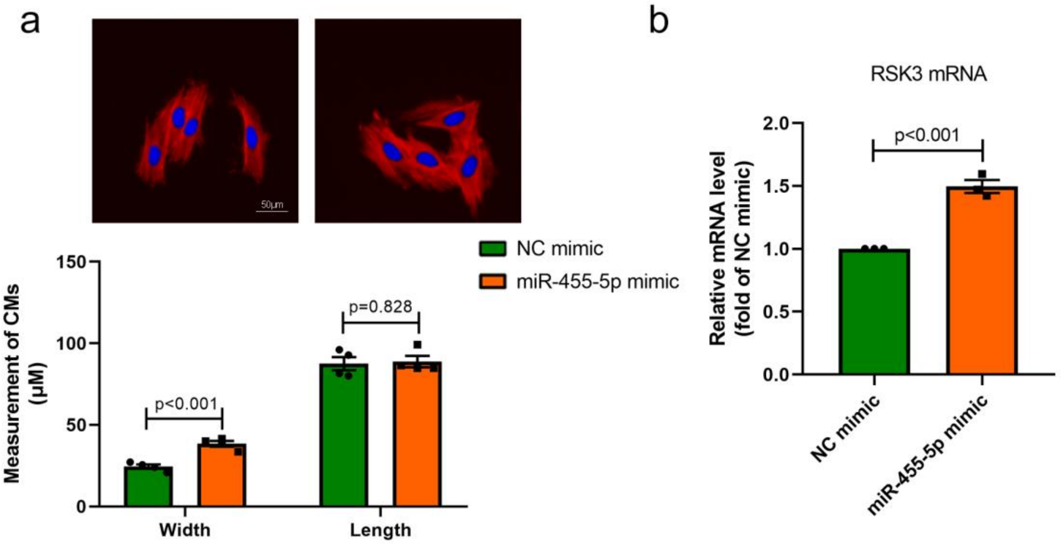
a
50μm
NC mimic
miR-455-5p mimic
Measurement of CMs (μM)
150
100
50
0
p<0.001
p=0.828
Width
Length
b
RSK3 mRNA
Relative mRNA level (fold of NC mimic)
2.0
1.5
1.0
0.5
0.0
p<0.001
NC mimic
miR-455-5p mimic

Fig. S10

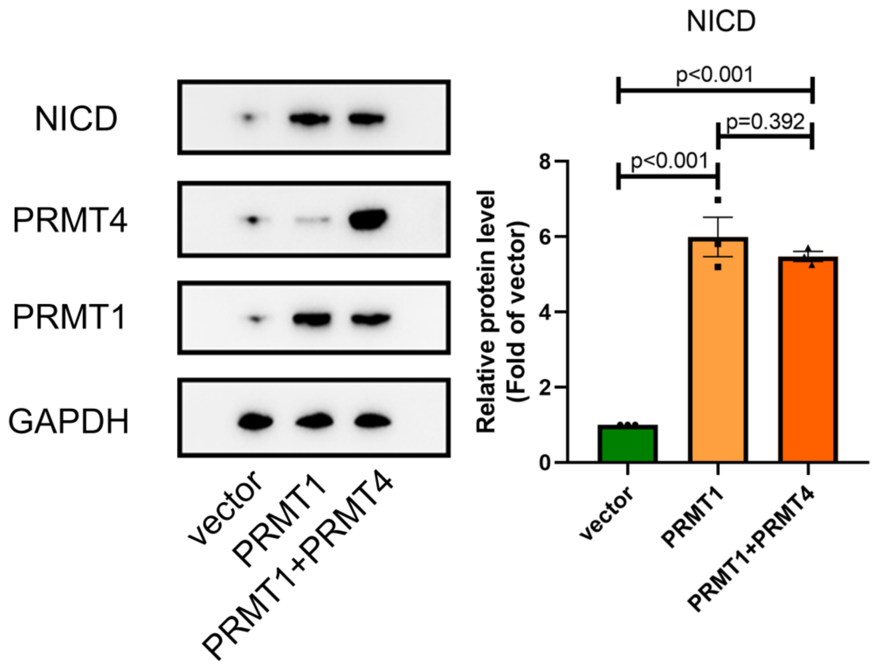

NICD
PRMT4
PRMT1
GAPDH
vector
PRMT1
PRMT1+PRMT4
NICD
p<0.001
p=0.392
p<0.001
Relative protein level
(Fold of vector)
8
6
4
2
0
vector
PRMT1
PRMT1+PRMT4
