# Supplementary material for: miR-455-5p promotes pathological cardiac remodeling via suppression of PRMT1-mediated Notch signaling pathway
Source: Cell Mol Life Sci. 2023 Nov 11;80(12):359. doi: 10.1007/s00018-023-04987-2 (PMC10640488; doi:10.1007/s00018-023-04987-2)
Supplement: Supplementary file 4 — Supplementary file4 (DOCX 28 KB) [file 18_2023_4987_MOESM4_ESM.docx]

**Table S1. Antibodies**

| **Antibody** | **Catalog number** | **Company** | **Source** | **Dilution** |
| --- | --- | --- | --- | --- |
| β-MHC | GTX100713-S | GeneTex | mouse | 1:1000 |
| Col1a | 95855S | Cell Signaling Technology | rabbit | 1:1000 |
| PRMT1 | GTX630186-S | GeneTex | rabbit | 1:1000 |
| GAPDH | 10494-1-AP 100UL | Proteintech | rabbit | 1:1000 |
| Notch1 | GT1224 | GeneTex | mouse | 1:1000 |
| NICD | 4147S | Cell Signaling Technology | rabbit | 1:1000 |
| RBP-Jκ | ab25949 | Abcam | rabbit | 1:1000 |
| pan-me2a | A20302-50 μL | Abclonal | rabbit | 1:500 |
| Lamin B1 | 66095-1-lg | Proteintech | mouse | 1:1000 |
| IgG | #2729 | Cell Signaling Technology | rabbit | 1:1000 |
| IgG | A0192 | Beyotime | mouse | 1:1000 |
| PRMT2 | A5835-50μL | Abclonal | rabbit | 1:1000 |
| PRMT3 | ab191562 | Abcam | rabbit | 1:1000 |
| PRMT4 | ab307091 | Abcam | rabbit | 1:1000 |
| PRMT6 | ab271091 | Abcam | rabbit | 1:1000 |
| PRMT8 | A07980-1-50ul | BOSTER | rabbit | 1:1000 |
| His | ABT2050 | Abbkine | mouse | 1:1000 |
| Presenilin 1 | 5643T | CST | rabbit | 1:1000 |

**Table S2. Primer of mRNA and miRNA**

| **Primer** | **Sequence** |  |
| --- | --- | --- |
| β-actin | Forward:5'- ACAACCTTCTTGCAGCTCCTC-3' |  |
|  | Reverse:5'-CTGACCCATACCCACCATCAC-3' |  |
| ANF | Forward:5'- CGTCTTGGCCTTTTGGCTTC-3' |  |
|  | Reverse:5'-GGTGGTCTAGCAGGTTCTTGAAA-3' |  |
| BNP | Forward:5'- GTTTGGGCTGTAACGCACT-3' |  |
|  | Reverse:5'-TCACTTCAAAGGTGGTCCCAG-3' |  |
| β-MHC | Forward:5'- AGCCTCAGCAGAGGAGTACA-3' |  |
|  | Reverse:5'-GGCTGAGCCTTGGATTCTCA-3' |  |
| Col1a | Forward:5'- GACAGCGTGGTGTGGTCGGT-3' |  |
|  | Reverse:5'-CACCAGGGGATCCCTCACGAC-3' |  |
| MMP1 | Forward:5'-TCCAGGCTTTATATGGGCCTTCC-3' |  |
|  | Reverse:5'-TCACATGGATGTGGTGTTGTTGC-3' |  |
| PRMT1 | Forward:5'-ACTGCATCATGGAGGTTTCCTG-3' |  |
|  | Reverse:5'-CATGGAGTTGCGGTACGTGA-3' |  |
| Notch1 | Forward:5'-AAAGAGGGCAGCAGCGG-3' |  |
|  | Reverse:5'-TGGGAGCATCTCAAGCCTCTT-3' |  |
| HES1 | Forward:5'-TCAACACGACACCGGACAAA-3' |  |
|  | Reverse:5'-GGAATGCCGGGAGCTATCTTT-3' |  |
| HEY1 | Forward:5'-GCAGTTAACTCCTCCCTGCC-3' |  |
|  | Reverse:5'-CGCCGAACTCAAGTTTCCATT-3' |  |
| HEY2 | Forward:5'-AGAACAATTACTCCGGGCACA-3' |  |
|  | Reverse:5'-TATTCGATCCCGACGCCTTT-3' |  |
| MYBPC3 | Forward:5'-GGCTTACTGTGGAACTGGCT-3' | |
|  | Reverse:5'-GGACTCAAAGATGTACCTGCC-3’ | |
| Desmin | Forward:5'-TCTCAAGGGCACCAACGAC-3’ | |
|  | Reverse:5'-GCGGGCCATCTCATCCTTTA-3’ | |
| PPARα | Forward:5'-TCGTGGAGTCCTGGAACTGA-3’ | |
|  | Reverse:5'-CTTCAGTCTTGGCTCGCCTC-3’ | |
| S6K1 | Forward:5'-CCTTGAGTATCTCAGTGGAGGAG-3’ | |
|  | Reverse:5'-CCCTTTTGATGTAAATGCCCCA-3’ | |
| Myh4 | Forward:5'-GAGAGAACAAGAATCTACAGCAGGA-3’ | |
|  | Reverse:5'-AGAGACGCCTGTAATTCACTCT-3’ | |
| Drp2 | Forward:5'-GCATCCCTGTCTGAGGGACTT-3’ | |
|  | Reverse:5'-ACTGTGGAGCATCAAACACCTTCT-3’ | |
| Tnnt2 | Forward:5'-TCCCTGACGGAGAGAGAGTG-3’ | |
|  | Reverse:5'-CCGACGCTTTTCGATCCTGT-3’ | |
| LDB3 | Forward:5'-CTGACCAGGCAGCAAGGAA-3’ | |
|  | Reverse:5'-CTGGGGTGATCCGGGAGA-3’ | |
| PRMT2 | Forward:5'-AACGCGGCTGAGACGTGA-3’ | |
|  | Reverse:5'-CAGCTGGTGCCTCCATAGTT-3’ | |
| PRMT3 | Forward:5'-CTGAGAACCACAAAGACAGCCA-3’ | |
|  | Reverse:5'-TTCCTTTTAAGGCTTCACCTGCT-3’ | |
| PRMT4 | Forward:5'-CCTGGCTCACACTACACGTC-3’ | |
|  | Reverse:5'-ACAATCCCTGTGTTGGCTAAGG-3’ | |
| PRMT6 | Forward:5'-TGCCGGTGGAACAAGATACA-3’ | |
|  | Reverse:5'-GATGCTGTCAGTGCCTTCCTAA-3’ | |
| PRMT8 | Forward:5'-ACCAAGTGCCACAAGAAAATGG-3’ | |
|  | Reverse:5'-TGAAATCGAGGTCTCGCACA-3’ | |
| MiR-455-5p | 5’-TGTGCCTTTGGACTACATCG-3’ | |
| U6 | 5’-TGGAACGCTTCACGAATTTGCG -3’ | |

**Table S3. Drugs**

| **Drug** | **Catalog number** | **Company** |
| --- | --- | --- |
| Isoproterenol hydrochloride | T1056 | TargetMol |
| CID2818500 | GC18577 | GLPBIO |

**Table S4. Reagents**

| **Reagent** | **Company** | **Catalog** |
| --- | --- | --- |
| Lipofectamine 2000 | Invitrogen | 11668019 |
| DAPI | Oneshine | FS1206-10mL |
| Anti-mouse IgG, HRP-linked secondary antibody | Cell Signaling Technology | #7076P2 |
| Anti-rabbit IgG, HRP-linked secondary antibody | Cell Signaling Technology | #7074P2 |
| rhodamine-phalloidin | GuangZhou Jiayan Biotech Co.,Ltd | 49734ES75 300T |
| protein A/G beads | Thermo Fisher | 20421 |
| RIPA lysis buffer | Beyotime | P0013B |
| BCA protein assay kit | Thermo Fisher | 23225 |
| Trizol | Invitrogen | 15596018 |
| RevertAid First Strand cDNA Synthesis Kit | Thermo Fisher | K1622 |
| THUNDERBIRD™ SYBR qPCR Mix | Toyobo | 622500 |
| miRNA 1st strand cDNA synthesis kit | Accurate Biotechnology | AG11716 |
| BIOG cfRNA Easy Kit | Changzhou Bio-generating Biotechnology | 51027 |
| Formaldehyde solution | Guangzhou Chemical Reagent Factory | 50-00-0 |
| IP lysis buffer | Beyotime | P0013 |
| Dual-Luciferase Reporter Assay System | Promega | E1910 |

**Table S5. Plasmid and small interference RNA**

| **Plasmid** | **Company** | **Location** | **Catelog Number** |
| --- | --- | --- | --- |
| PRMT1 | VIC GENE | Guangzhou, China | G8152 |
| PcDNA3.1+ | Vigenebio | Shandong, China | G107083-1 |
| Wild type Notch1 | Shhebio | Shanghai, China | P53055 |
| Mutant type Notch1 (R1748A, R1750A, R1751A, R1752A) | Shhebio | Wuhan, China | MNM_001105721 |
| pCMV vector | MiaoLing | Wuhan, China | P0818 |
| Wild type PRMT1 3’UTR Dual Luciferase Reporter Plasmid | Guangzhou RiboBio Co., Ltd. | Guangzhou, China | ZT003 |
| mutant PRMT1 3’UTR Dual Luciferase Reporter Plasmid | Guangzhou RiboBio Co., Ltd. | Guangzhou, China | ZT003 |
| **siRNA** | **Company** | **Target sequence** | **Catelog Number** |
| si-PRMT1 | Guangzhou RiboBio Co., Ltd. | GCTCCAGTATCTCTGATTA | siBDM1999A |
| miR-455-5p mimic | Guangzhou RiboBio Co., Ltd. | UAUGUGCCUUUGGACUACAUCG | PA20210817016 |
| miR-455-5p inhibitor | Guangzhou RiboBio Co., Ltd. | CGAUGUAGUCCAAAGGCACAUA | miR20000-5 |
| miR-455-5p agomir | Guangzhou RiboBio Co., Ltd. | UAUGUGCCUUUGGACUACAUCG | miR40003485-160 |
| miR-455-5p antagomir | Guangzhou RiboBio Co., Ltd. | CGAUGUAGUCCAAAGGCACAUA | miR30003485-640 |
| NC mimic | Guangzhou RiboBio Co., Ltd. | - | miR1N0000001-1-5 |
| NC inhibitor | Guangzhou RiboBio Co., Ltd. | - | ssR180425043707 |
| NC agomir | Guangzhou RiboBio Co., Ltd. | - | miR4N0000001-160 |
| NC antagomir | Guangzhou RiboBio Co., Ltd. | - | miR3N0000001-640 |
