# Supplementary material for: miR-455-5p promotes pathological cardiac remodeling via suppression of PRMT1-mediated Notch signaling pathway
Source: Cell Mol Life Sci. 2023 Nov 11;80(12):359. doi: 10.1007/s00018-023-04987-2 (PMC10640488; doi:10.1007/s00018-023-04987-2)
Supplement: Supplementary file 5 — Supplementary file5 (DOCX 24 KB) [file 18_2023_4987_MOESM5_ESM.docx]

**Table S6. The top ranking genes regulated by PRMT1**

**(Top 20)**

| **Gene name** | **Fold change** | **P value** | **Description** |
| --- | --- | --- | --- |
| ***Myh4*** | 0.41017174 | 2.73E-23 | myosin heavy chain 4 |
| Scd | 1.583481487 | 3.91E-10 | stearoyl-CoA desaturase |
| Lbh | 0.496890547 | 7.42E-10 | LBH regulator of WNT signaling pathway |
| Insig1 | 1.579754089 | 8.80E-10 | insulin induced gene 1 |
| Lmcd1 | 0.561905723 | 6.55E-09 | LIM and cysteine-rich domains 1 |
| Cacna1s | 0.447667658 | 1.05E-08 | calcium voltage-gated channel subunit alpha1 S |
| ***Drp2*** | 0.059333883 | 8.09E-08 | dystrophin related protein 2 |
| LOC100910418 | 0.20381046 | 1.78E-07 | tissue-type plasminogen activator-like |
| E2f2 | 1.783609761 | 2.08E-07 | E2F transcription factor 2 |
| Casq2 | 0.710890864 | 3.24E-07 | calsequestrin 2 |
| ***Tnnt2*** | 0.474769776 | 5.44E-07 | troponin T2, cardiac type |
| Fmod | 0.485519115 | 8.09E-07 | fibromodulin |
| LOC100910446 | 17.14005698 | 1.01E-06 | syntaxin-7-like |
| Lamc2 | 0.663054793 | 1.02E-06 | laminin subunit gamma 2 |
| Sfrp2 | 91.22769749 | 1.69E-06 | secreted frizzled-related protein 2 |
| Ephx1 | 0.62420854 | 1.94E-06 | epoxide hydrolase 1 |
| ***Ldb3*** | 0.554477183 | 2.78E-06 | LIM domain binding 3 |
| Gsta1 | 0.457993808 | 3.69E-06 | glutathione S-transferase alpha 1 |
| Cox6a2 | 0.481263862 | 4.46E-06 | cytochrome c oxidase subunit 6A2 |
| Efnb2 | 1.471614708 | 4.54E-06 | ephrin B2 |

**Table S7. Univariable Logistic Regression for Factors To discriminate Non-cLVH and cLVH**

|  | **Significance**  **(p value)** | **OR**  **(Odds ratio)** | **95% CI**  **For OR** |
| --- | --- | --- | --- |
| BMI, kg/m2 | 0.750 | 0.974 | [0.830, 1.144] |
| Age | 0.374 | 1.022 | [0.975, 1.071] |
| sex (Male) | 0.449 | 0.606 | [0.166, 2.215] |
| △miR-455-5p | ***0.002***** | ***3.382*** | ***[1.540, 7.431]*** |
| Diabetes | 0.106 | 2.877 | [0.800, 10.350] |
| Coronary artery disease | 0.473 | 2.308 | [0.236, 22.598] |
| Valvular heart disease | 0.262 | 4.143 | [0.346, 49.657] |
| SBP, mmHg | 0.211 | 1.017 | [0.991, 1.043] |
| DBP, mmHg | 0.211 | 0.974 | [0.936, 1.015] |
| LVEF, % | 0.092 | 1.055 | [0.991, 1.123] |

BMI, body mass index; SBP, systolic blood pressure; DBP, diastolic blood pressure; LVEF, left ventricular ejection fraction;

△miR-455-5p, log10 (concentration of miR-455-5p in 100ml blood); CI, confidential interval

**P<0.01.

**Table S8. Multivariable Logistic Regression for Factors To discriminate Non-cLVH and cLVH**

|  | **Significance**  **(p value)** | **OR**  **(Odds ratio)** | **95% CI**  **For OR** |
| --- | --- | --- | --- |
| BMI, kg/m2 | 0.882 | 1.015 | [0.831, 1.241] |
| Age | 0.350 | 1.039 | [0.959, 1.127] |
| Sex (Male) | 0.083 | 0.129 | [0.013, 1.310] |
| △miR-455-5p | ***0.006***** | 5.208 | [1.592, 17.034] |

BMI; body mass index; △miR-455-5p, log10 (concentration of miR-455-5p in 100ml blood); CI, confidential interval;

^**^P<0.01.

**Table S9. Other echocardiography data of the mice in NC agomir and miR-455-5p agomir**

|  | NC agomir | MiR-455-5p agomir | P value |
| --- | --- | --- | --- |
| LVEDV (μl) | 44 ± 10 | 52 ± 8 | 0.163 |
| LVESV (μl) | 13 ± 3 | 21 ± 10 | 0.066 |
| SV (μl) | 31 ± 8 | 30 ± 7 | 0.911 |
| HR (bpm) | 409 ± 29 | 309 ± 81 | **0.018** |
| CO (μl/min) | 13430 ± 1314 | 8942 ± 1054 | **0.012** |

Abbreviation: LVEDV, left ventricular end diastolic volume; LVESV, left ventricular end systolic volume; SV, stroke volume; HR, heart rate; CO, cardiac output.

Data were presented as mean ± SD.
